# Indole Derivatives: A Versatile Scaffold in Modern Drug Discovery—An Updated Review on Their Multifaceted Therapeutic Applications (2020–2024)

**DOI:** 10.3390/molecules29194770

**Published:** 2024-10-09

**Authors:** Xingyou Mo, Devendra Pratap Rao, Kirandeep Kaur, Roket Hassan, Ahmed S. Abdel-Samea, Sara Mahmoud Farhan, Stefan Bräse, Hamada Hashem

**Affiliations:** 1 School of Engineering, Guangzhou College of Technology and Business, Guangzhou 510850, China; 2 Coordination Chemistry Laboratory, Department of Chemistry, Dayanand Anglo-Vedic (PG) College, Kanpur 208001, Uttar Pradesh, India; 3 Department of Chemistry, Maharaja Ranjit Singh Punjab Technical University, Bathinda 151001, Punjab, India; 4 Department of Pharmaceutical Chemistry, Faculty of Pharmacy, Sohag University, Sohag 82524, Egypt; 5 Pharmacology & Toxicology Department, Faculty of Pharmacy, Deraya University, New Minia 61768, Egypt; 6 Department of Microbiology and Immunology, Faculty of Pharmacy, Deraya University, New Minia 61768, Egypt; 7 Institute of Biological and Chemical Systems—Functional Molecular Systems (IBCS-FMS), Karlsruhe Institute of Technology (KIT), Kaiserstrasse 12, 76131 Karlsruhe, Germany

**Keywords:** indole, biological activities, anticancer, antimicrobial, anti-inflammatory, antidiabetic, neurodegenerative diseases, antihypertensive

## Abstract

Indole derivatives have become an important class of compounds in medicinal chemistry, recognized for their wide-ranging biological activities and therapeutic potential. This review provides a comprehensive overview of recent advances in the evaluation of indole-based compounds in the last five years, highlighting their roles in cancer treatment, infectious disease management, anti-inflammatory therapies, metabolic disorder interventions, and neurodegenerative disease management. Indole derivatives have shown significant efficacy in targeting diverse biological pathways, making them valuable scaffolds in designing new drugs. Notably, these compounds have demonstrated the ability to combat drug-resistant cancer cells and pathogens, a significant breakthrough in the field, and offer promising therapeutic options for chronic diseases such as diabetes and hypertension. By summarizing recent key findings and exploring the underlying biological mechanisms, this review underscores the potential of indole derivatives in addressing major healthcare challenges, thereby instilling hope and optimism in the field of modern medicine.

## 1. Introduction

**Figure 1:**
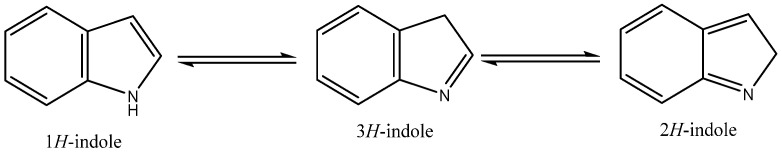
Tautomeric structures of the indole ring.

The indole core, a weakly basic molecule consisting of a pyrrole ring fused to a benzene ring, is known for its aromatic nature due to the delocalization of ten π-electrons. Indole can exist in three tautomeric forms—1*H*-indole, 2*H*-indole, and 3*H*-indole (Figure 1) [1]—which differ in the position of the hydrogen atom within the ring structure. These tautomers can influence the chemical reactivity and biological interactions of indole derivatives. Indole derivatives have long fascinated researchers due to their diverse biological activities and therapeutic potential [2,3,4,5]. As core structures in many natural products and pharmaceuticals, indoles are pivotal scaffolds in drug discovery and development. This review aims to provide an updated overview of recent progress in the biological activities of indole derivatives, highlighting their multifaceted roles in various therapeutic areas from 2020 to 2024.

Historically, indole derivatives have been central to developing several important drugs. For instance, the indole alkaloid reserpine derived from the *Rauwolfia* plant has been used as an antihypertensive [6] and antipsychotic agent [7]. The structural significance of indoles is further underscored by their presence in essential biomolecules such as serotonin and melatonin. Serotonin, a neurotransmitter with a critical role in regulating mood, appetite [8], and sleep [9], features an indole moiety, which is essential for its biological activity [10]. Similarly, melatonin, another indole-based compound, regulates circadian rhythms [11] and has been investigated for its potential in treating sleep disorders [12] and as an antioxidant [11].

**Figure 2:**
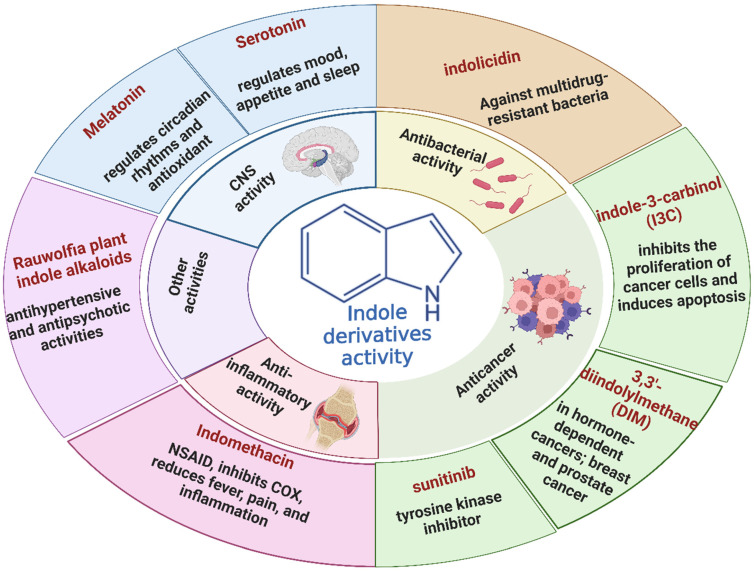
Different natural and commercially available indole derivatives and their biological activities.

In recent years, significant attention has been directed toward the anticancer properties of indole derivatives [13]. Compounds such as indole-3-carbinol (I3C), found in cruciferous vegetables like broccoli and Brussels sprouts, have demonstrated the ability to inhibit the proliferation of cancer cells and induce apoptosis. I3C and its dimeric product, 3,3′-diindolylmethane (DIM), have been shown to modulate estrogen metabolism and exhibit anticancer effects in hormone-dependent cancers such as breast and prostate cancer [14]. Furthermore, synthetic indole derivatives like sunitinib, a tyrosine kinase inhibitor, have been effectively utilized as targeted therapies in renal cell carcinoma [15] and gastrointestinal stromal tumors [16], showcasing their efficacy in oncology. The versatility of indole scaffolds in interacting with various biological targets, such as kinases, has made them attractive candidates for anticancer drug development, intriguing and engaging researchers in the field (Figure 2).

Beyond oncology, the therapeutic potential of indole derivatives extends to addressing the growing challenge of bacterial resistance [17]. The emergence of antibiotic-resistant strains, such as methicillin-resistant *Staphylococcus aureus* (MRSA) and carbapenem-resistant *Enterobacteriaceae*, has necessitated the development of new antimicrobial agents. Indole-based compounds, including indolicidin—a cationic antimicrobial peptide derived from bovine neutrophils [18]—exhibit potent antibacterial activity against various pathogens, including multidrug-resistant bacteria. The ability of indole derivatives to disrupt bacterial membranes and inhibit biofilm formation [19] has opened new avenues for their application as antimicrobial agents.

Moreover, the anti-inflammatory properties of indole derivatives have been explored extensively. Indomethacin, a nonsteroidal anti-inflammatory drug (NSAID) containing an indole structure, has been widely used to reduce fever, pain, and inflammation [20]. Recent research has identified novel indole derivatives that modulate key inflammatory pathways, such as the NF-κB [21] and COX-2 (Cycloxygenase-2) [22] pathways, offering potential therapeutic options for chronic inflammatory diseases like rheumatoid arthritis and inflammatory bowel disease. Additionally, the dual inhibition of COX and LOX (Lipoxygenase) enzymes by certain indole derivatives has been proposed as a strategy to reduce the gastrointestinal side effects [23] commonly associated with traditional NSAIDs. Numerous commercially available drugs containing indole scaffold, as illustrated in Table 1, highlight the wide-ranging therapeutic applications of this versatile scaffold.

The structural versatility of indole scaffolds, characterized by their ability to participate in various chemical reactions and form diverse chemical bonds, underpins their widespread use in medicinal chemistry. The incorporation of different substituents on the indole ring has been shown to significantly alter the biological activity of these compounds, leading to the discovery of novel drugs with improved efficacy and safety profiles. Recent years have witnessed advancements in understanding indole derivatives’ structure–activity relationships (SAR), particularly in optimizing drug-like properties such as solubility, permeability, and metabolic stability. Exploring indole derivatives in other therapeutic areas, such as neurodegenerative diseases, cardiovascular disorders, and diabetes, further highlights their potential as lead compounds for developing new drugs.

This review aims to provide an updated and comprehensive overview of the recent progress in the biological activities of indole derivatives from 2020 to 2024. By focusing on their multifaceted therapeutic applications and the ongoing research in this dynamic field, we aim to underscore the potential of indole derivatives as versatile scaffolds in modern drug discovery for novel therapies.

## 2. Biological Activities of Indole Derivatives

### 2.1. Anticancer Activity

**Figure 3:**
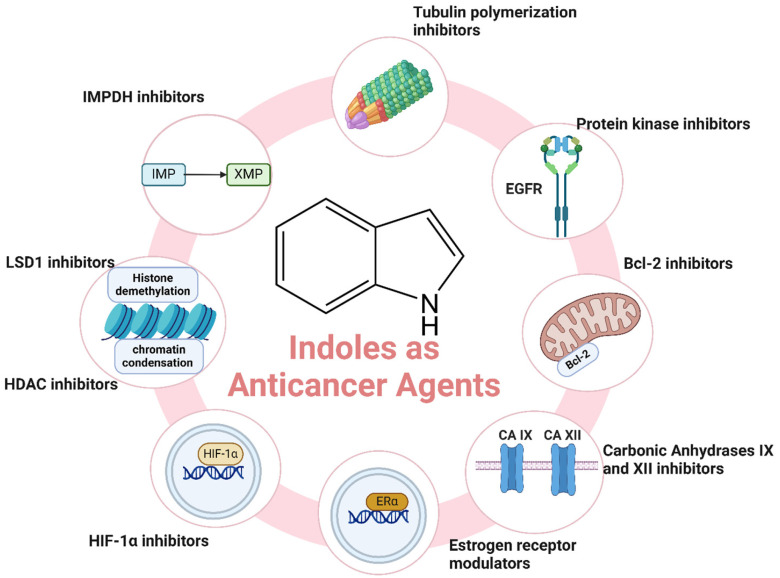
Different mechanisms of indole derivatives as anticancer agents.

Cancer remains one of the leading causes of morbidity and mortality worldwide, with an estimated 19.3 million new cases and nearly 10 million cancer-related deaths reported globally in 2020 [39,40,41,42,43]. The disease’s burden is expected to rise significantly in the coming decades, driven by aging populations and increasing exposure to risk factors such as tobacco use, unhealthy diet, and environmental pollutants [44]. Despite advances in cancer treatment, including surgery, radiation, and chemotherapy, the need for more effective and targeted therapies is paramount [45,46,47]. Indole derivatives have emerged as promising anticancer agents due to their ability to act on different key biological targets such as tubulin [48], protein kinases [49], HDAC (Histone Deacetylase) [50], etc. (Figure 3). The structural versatility of indoles allows for the design of compounds that can specifically target cancer cells while minimizing toxicity to normal tissues. Here, we highlight recent advancements and key examples of indole derivatives demonstrating significant anticancer activity.

#### 2.1.1. Tubulin Polymerization Inhibitors

Inhibiting tubulin polymerization is a crucial strategy in cancer treatment because it directly interferes with microtubule dynamics, essential for proper cell division and proliferation [51,52,53]. Microtubules are vital for forming the mitotic spindle, and their disruption leads to cell cycle arrest at the metaphase, ultimately causing apoptosis in cancer cells, which often have high mitotic rates [40,54]. Indole derivatives, such as vinca alkaloids (e.g., vinblastine and vincristine), are key anticancer agents known to inhibit tubulin polymerization [55]. These compounds bind to tubulin and prevent its assembly into microtubules, effectively stopping the cell division process in cancerous cells. Beyond vinca alkaloids, indole-based compounds have shown promise as potential anticancer agents due to their ability to target tubulin and other related pathways involved in cell proliferation [48].

**Figure 4:**
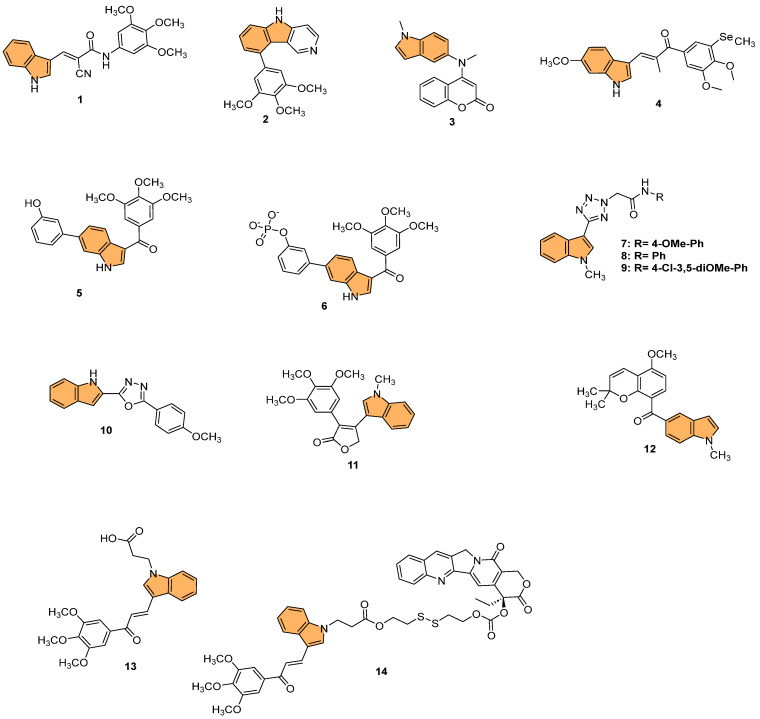
Structures of compounds **1**−**14** as tubulin polymerization inhibitors.

Building on this, recent studies have explored the development of novel indole derivatives with enhanced anticancer properties. For instance, Hawash et al. synthesized a series of novel substituted indole-acrylamide derivatives and evaluated their anticancer activities as potential tubulin-targeting agents [56]. Out of these, **1** (Figure 4) emerged as the most potent, showing significant tubulin polymerization inhibitory activity with an IC_50_ of 5.0 µM against Huh7 hepatocellular carcinoma cells. Compound **1** induced G2/M-phase cell cycle arrest in Huh7 cells, leading to apoptotic cell death. The structure–activity relationship analysis indicated that the presence of a cyano group on the prop-2-en-1-on linker enhanced the compound’s potency. Molecular docking studies revealed that compound **1** forms hydrogen bonds with βAsn258 and βCys241 in the colchicine-binding site of tubulin, stabilizing the interaction. The study highlights the potential of indole-acrylamide derivatives as promising candidates for developing tubulin-targeting anticancer therapies.

Furthermore, Shi et al. designed and synthesized a series of 9-aryl-5H-pyrido [4,3-b] indole derivatives as potential tubulin polymerization inhibitors [57], evaluating their antitumor activities. Their efforts culminated in the discovery of compound **2** (Figure 4), which demonstrated the most potent antiproliferative activity against HeLa cells with an IC_50_ value of 8.7 µM. This compound effectively inhibited tubulin polymerization and disrupted the microtubule network, as confirmed by immunofluorescence staining. Mechanistic studies revealed that **2** arrested the cell cycle in the G2/M phase and induced apoptosis in a dose-dependent manner. Molecular docking studies showed that **2** binds to the colchicine-binding site of tubulin, forming stable interactions with key residues such as Asnβ258 and Valβ238. The cumulative evidence points to compound **2** as a promising lead for developing new anticancer agents targeting tubulin.

In another approach, Song et al. designed and synthesized novel coumarin-indole derivatives and evaluated their efficacy as tubulin polymerization inhibitors targeting the colchicine-binding site [58]. Results showed that compound **3** (Figure 4) demonstrated the most potent anticancer activity, particularly against the gastric cancer cell line MGC-803, with an IC_50_ value of 0.011 μM. Compound **3** also exhibited strong inhibitory activity across a range of cancer cell lines, with IC_50_ values below 0.1 μM for 17 other cancer lines and less than 0.05 μM for 8 lines. The compound effectively inhibited tubulin polymerization with an IC_50_ of 2.46 μM and showed potent suppression of MAPK pathway kinases in cancer progression. In vivo studies using a mouse xenograft model of MGC-803 cells demonstrated significant tumor growth inhibition (TGI) of 70% and 80% at doses of 15 mg/kg and 30 mg/kg, respectively, without notable toxicity. The findings indicate that **3** is a promising candidate for further development as a tubulin-targeting anticancer agent.

Moreover, Yan et al. synthesized indole-chalcone derivatives, modifying their structures to create dual-targeted agents that inhibit tubulin polymerization and thioredoxin reductase (TrxR) [59]. In the study, compound **4** (Figure 4) exhibited the most potent antiproliferative activity against six human cancer cell lines, with IC_50_ values ranging from 6 to 35 nM. This compound showed superior efficacy in inhibiting tubulin polymerization with an IC_50_ of 0.81 µM and acted as a TrxR inhibitor with an IC_50_ of 3.728 µM. Mechanistically, **4** caused cell cycle arrest at the G2/M phase and induced apoptosis by disrupting mitochondrial function and increasing reactive oxygen species (ROS) levels. The in vivo studies in a xenograft model of MHCC-97H cells showed that **4** significantly inhibited tumor growth by 75.4% without causing notable toxicity. Therefore, compound **4** holds promise as a dual-targeted anticancer agent, offering an effective strategy to combat drug-resistant tumors.

In a related effort, Ren et al. designed and synthesized a series of 6-aryl-3-aroyl-indole analogs as inhibitors of tubulin polymerization [60], inspired by colchicine and combretastatin A-4 (CA4). Among the analogs, **5** (Figure 4) exhibited the most potent activity, with an IC_50_ of 0.57 μM for tubulin polymerization inhibition and significant cytotoxicity against MDA-MB-231 breast cancer cells (IC_50_ = 102 nM). Compound **5** induced G2/M phase arrest, disrupted microtubule structures, and inhibited cell migration in a scratch assay. Additionally, the compound bound effectively to the colchicine site on tubulin, as confirmed by molecular docking studies. The phosphate prodrug of **5**, compound **6** (Figure 4), demonstrated vascular disrupting activity (VDA) in vivo, reducing bioluminescence signal in a kidney cancer model in mice by over 90% within 2.5 h. The study highlights **5** and its prodrug as promising candidates for tubulin-targeting cancer therapies.

Reddy et al. synthesized a series of indole-tetrazole coupled aromatic amides and evaluated their in vitro anticancer activity, tubulin polymerization inhibition [61], and molecular docking studies. Compounds **7**, **8**, and **9** (Figure 4) exhibited significant anticancer activity, with IC_50_ values ranging from 3.5 to 8.7 μM against MCF-7 (breast), A549 (lung), and SKOV3 (ovarian) cancer cell lines, outperforming the standard drug etoposide. Compounds **7** and **9** demonstrated enhanced tubulin polymerization inhibition, with IC_50_ values of 0.52 and 0.34 μM, respectively, compared to the standard combretastatin A-4 (IC_50_ = 1.12 μM). Molecular docking studies revealed that these compounds showed strong binding interactions with α,β-tubulin, with compound **9** forming a hydrogen bond with ASN329. The ADMET properties of the active compounds indicated favorable drug-like profiles, adhering to Lipinski and other rules, making them promising candidates for further development as anticancer agents targeting tubulin polymerization.

Similarly, Boda et al. synthesized a series of indole-1,3,4-oxadiazole hybrids and evaluated their anticancer activity and ability to inhibit tubulin polymerization [62]. They identified compound **10** (Figure 4) as the most potent, with IC_50_ values of 4.2 µM, 3.1 µM, and 2.1 µM against SKOV3 (ovarian), A549 (lung), and MCF-7 (breast) cancer cell lines, respectively. Compound **10** also demonstrated superior tubulin polymerization inhibition with an IC_50_ of 0.48 µM, surpassing the standard combretastatin A-4 (IC_50_ = 1.13 µM). Molecular docking studies revealed that **10** forms stable interactions with α- and β-tubulin, displaying the highest binding energy of −8.54 kcal/mol and forming hydrogen bonds with key residues (PRO175, VAL177, and SER174). This proves that indole-1,3,4-oxadiazole hybrids, particularly **10**, are promising candidates for further development as tubulin-targeting anticancer agents.

Likewise, Hurysz et al. developed a series of indole-substituted furanones and assessed their potential as anti-tubulin agents with broad anticancer activity [63]. Notably, compound **11** (Figure 4) emerged as the most potent, displaying significant cytotoxicity against U-937 cancer cells with an EC_50_ value of 0.6 μM. The compound effectively inhibited tubulin polymerization, a mechanism similar to the well-known colchicine inhibitor. Molecular docking studies confirmed that **11** binds favorably to the colchicine-binding site (CBS) on tubulin, forming a hydrogen bond with V181 at a distance of 2.39 Å, which is notably stronger than the corresponding bond in colchicine (3.05 Å). This strong binding affinity likely contributes to its enhanced biological activity. Furthermore, **11** exhibited broad-spectrum anticancer efficacy, as demonstrated by its performance in the NCI-60 cell line assay where it achieved GI_50_ values as low as 28 nM against MCF7 breast cancer cells. This evidence points to compound **11** as a highly promising candidate for advancing novel anticancer treatments that target tubulin polymerization.

In a similar study, Liang et al. synthesized novel indole-containing hybrids derived from millepachine and evaluated their antitumor activities [64]. The most potent compound, **12** (Figure 4), exhibited significant cytotoxicity across five human cancer cell lines, with IC_50_ values ranging from 0.022 to 0.074 μM, which is around 100 times more potent than millepachine. Compound **12** inhibited tubulin polymerization with an IC_50_ of 2.07 μM and induced G2/M cell cycle arrest in cancer cells. Furthermore, it triggered apoptosis through reactive oxygen species (ROS) accumulation and mitochondrial membrane potential (MMP) disruption. Notably, **12** displayed lower toxicity toward normal human cells and maintained efficacy against drug-resistant cancer cell lines, highlighting its potential as a promising antitumor agent.

Wang et al. synthesized a series of GSH-responsive prodrugs derived from indole-chalcone and camptothecin (CPT), aiming to overcome multidrug resistance (MDR) in cancer therapy [65]. The most potent compound, **14** (Figure 4), displayed superior antiproliferative activity compared to CPT alone, with IC_50_ values as low as 0.15 μM against HCT-116 colon cancer cells. Notably, **14** exhibited potent efficacy against paclitaxel-resistant (PTX-resistant) HCT-116 cells, with an IC_50_ of 0.25 μM, significantly outperforming both CPT and a physical mixture of **13** (Figure 4) (an indole-chalcone derivative) and CPT. Mechanistic studies revealed that **14** acts as a GSH-responsive prodrug, releasing CPT and **13** upon activation by GSH, leading to inhibition of tubulin polymerization, mitochondrial membrane depolarization, and the induction of apoptosis. Additionally, **14** triggered autophagy and enhanced intracellular reactive oxygen species (ROS) levels, further promoting cell death. In vivo, **14** significantly inhibited tumor growth in PTX-resistant colorectal cancer xenograft models, with a tumor growth inhibition rate of 64.7%, while displaying minimal toxicity toward healthy cells. With its strong activity, compound **14** emerges as a promising candidate for overcoming MDR in colorectal cancer therapy.

#### 2.1.2. Protein Kinase Inhibitors

Protein kinase inhibitors (PKIs) are a class of enzyme inhibitors that block the activity of protein kinases, which are enzymes responsible for adding phosphate groups to proteins (a process known as phosphorylation) [66]. These enzymes are crucial for cell signaling pathways that regulate cell growth and survival [67]. By inhibiting these kinases, PKIs can effectively disrupt cancer cell proliferation with fewer side effects than traditional chemotherapy [68]. EGFR inhibitors are a key example of PKIs, targeting the epidermal growth factor receptor (EGFR). This receptor is often overexpressed or mutated in cancers like non-small cell lung cancer (NSCLC). Drugs such as erlotinib and gefitinib block EGFR activity, preventing uncontrolled cell division and offering a more personalized treatment [69].

**Figure 5:**
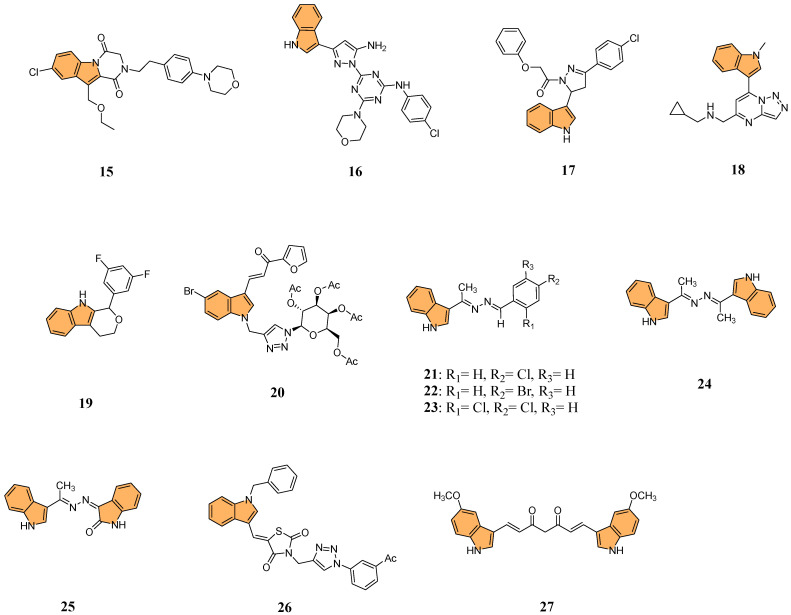
Structures of compounds **15**–**27** as protein kinase inhibitors.

Building on the potential of indole derivatives as PKIs, Gomaa et al. synthesized a series of novel 2,3-dihydropyrazino [1,2-a]indole-1,4-dione derivatives. They evaluated their efficacy as dual inhibitors of EGFR and BRAFV600E [70], with promising antiproliferative and antioxidant activities. Compound **15** (Figure 5) was particularly notable for its potent activity, exhibiting superior EGFR inhibition with an IC_50_ value of 32 nM compared to the reference drug erlotinib (IC_50_ = 80 nM). Additionally, compound **15** demonstrated potent BRAFV600E inhibitory activity, with an IC_50_ value of 45 nM, alongside significant antiproliferative activity against cancer cell lines, showing a GI_50_ value of 35 nM. This compound also displayed cytotoxicity against the LOX-IMVI melanoma cell line, with an IC_50_ of 1.02 μM, and exhibited strong antioxidant activity, comparable to Trolox, with a DPPH (2,2-diphenyl-1-picrylhydrazyl) radical scavenging rate of 72.7% at 10 μM. Compound **15** induced apoptosis and G2/M cell cycle arrest, marking it a potent candidate for further development as an anticancer agent targeting EGFR and BRAFV600E.

In another investigation, Shawish et al. designed and synthesized a series of pyrazolyl-s-triazine compounds incorporating an indole motif, aiming to develop dual inhibitors of EGFR and CDK-2 for anticancer therapy [71]. The most prominent activity was observed with compound **16** (Figure 5), which showed remarkable cytotoxicity against A549 lung cancer cells with an IC_50_ value of 2.66 μM. It also exhibited potent inhibition of EGFR, with an IC_50_ value of 34.1 nM, significantly outperforming the reference drug erlotinib (IC_50_ = 67.3 nM). Furthermore, **16** showed robust CDK-2 inhibition, achieving 91.4% inhibition at 10 μM while inhibiting EGFR by 93.6% at the same concentration. The compound effectively promoted apoptosis in lung cancer cells, increasing total apoptosis by 71.6-fold compared to the control, with 43.7% apoptosis versus 0.61% for untreated cells. With its dual-target inhibition of EGFR and CDK-2 and significant pro-apoptotic effects, compound **16** represents a highly promising candidate for further development in anticancer therapies.

In 2022, Khalilullah et al. synthesized new pyrazolinyl-indole derivatives and evaluated their anticancer potential, particularly as EGFR inhibitors [72]. The study revealed that compound **17** (Figure 5) displayed the most significant anticancer activity across multiple cancer cell lines, achieving 78.76% growth inhibition in leukemia cells at a concentration of 10 μM, significantly outperforming the standard reference drug imatinib (9% inhibition). The study confirmed the cytotoxic activity of **17** in nine cancer cell line panels, including leukemia, breast, and colon cancers. Molecular docking studies revealed that **17** strongly interacts with the active site of the EGFR tyrosine kinase, forming hydrogen bonds with key residues such as Met793, enhancing its inhibitory effect.

Yu et al. designed and synthesized a series of [1,2,4]triazolo [1,5-a]pyrimidine indole derivatives to evaluate their antiproliferative activities against various cancer cell lines, particularly targeting the ERK signaling pathway [73] in gastric cancer cells (MGC-803). The ERK signaling pathway, known as the extracellular signal-regulated kinase pathway, regulates various cellular processes, including proliferation, differentiation, and survival [74]. Aberrant activation of this pathway is frequently observed in many types of cancer [75], contributing to uncontrolled cell growth and resistance to apoptosis. The most potent compound, **18** (Figure 5), exhibited strong antiproliferative effects with IC_50_ values of 9.47 μM against MGC-803, 9.58 μM against HCT-116, and 13.1 μM against MCF-7 cells, outperforming the standard drug 5-Fu. Compound **18** significantly inhibited the ERK signaling pathway, reducing the phosphorylation levels of key proteins including ERK1/2, c-Raf, MEK1/2, and AKT—in a dose-dependent manner. Additionally, **18** induced G2/M phase arrest and apoptosis in MGC-803 cells by upregulating pro-apoptotic proteins like Bax and cleaved-Caspase7 while downregulating anti-apoptotic proteins such as Mcl-1 and Bcl-2. Given its potent activity, compound **18** could be a valuable candidate for anticancer drug development targeting the ERK signaling pathway.

The PI3K/AKT/mTOR pathway is a critical signaling pathway in cancer, regulating cell growth, proliferation, and survival. Dysregulation of this pathway is commonly observed in various cancers, making it a significant target for anticancer therapies [76,77]. Building on this, Qin et al. synthesized a series of 1,3,4,9-tetrahydropyrano [3,4-b]indoles and evaluated their potential as treatments for triple-negative breast cancer (TNBC) by targeting the PI3K/AKT/mTOR pathway[78]. The research showed that compound **19** (Figure 5) demonstrated the most potent anticancer activity against MDA-MB-231 cells with an IC_50_ value of 2.29 μM. Mechanistic studies revealed compound **19** induced G0/G1 cell cycle arrest, triggered apoptosis, and disrupted mitochondrial membrane potential (MMP). Additionally, it increased reactive oxygen species (ROS) levels, reduced intracellular glutathione (GSH), elevated intracellular calcium ions (Ca^2+^), and activated caspase cascades. Compound **19** significantly suppressed key regulators of the PI3K/AKT/mTOR pathway in MDA-MB-231 cells, including reduced phosphorylation of AKT and mTOR. With its ability to selectively target cancer cells and disrupt critical survival pathways, compound **19** stands out as a promising lead for the treatment of TNBC.

Tyagi et al. developed a series of indole-chalcone-based glycohybrids using click chemistry to explore their anticancer potential. They synthesized three distinct glycohybrids using D-glucose, D-galactose, and D-mannose, linking these sugars to indole-chalcone derivatives via a 1,2,3-triazole bridge [79]. The research identified **20** (Figure 5) as the most potent anticancer compound, particularly against MCF-7 breast cancer cells, with an impressive IC_50_ value of 1.05 μM and a high selectivity index (SI > 161), indicating strong selectivity for cancer cells over normal cells. It also showed considerable cytotoxicity against MDA-MB-231 cells with an IC_50_ of 18.03 μM. Compound **20** exhibited no significant toxicity toward normal MCF-10A cells, highlighting its selectivity. Molecular docking studies also revealed that **20** binds effectively to the HCK protein target, with a docking score of −13.42 kcal/mol and higher binding affinity than the natural HCK inhibitor PP1 (−6.77 kcal/mol).

Ibrahim et al. synthesized a series of azine derivatives bearing indole moieties using a green mechanochemical process and evaluated their anticancer activity against HCT-116 (colon), HepG2 (liver), and MCF-7 (breast) cancer cell lines [80]. Compounds **21**, **22**, **23**, **24**, and **25** (Figure 5) exhibited potent cytotoxic activity with IC_50_ values ranging from 4.27 to 8.15 µM against HCT-116, compared to the reference drug doxorubicin (IC_50_ = 5.23 µM). Similarly, these compounds demonstrated significant activity against HepG2, with IC_50_ values of 4.09–9.05 µM, and against MCF-7, with IC_50_ values of 6.19–8.39 µM. Molecular docking studies revealed that compound **24** showed the highest binding affinity toward CDK-5 with a docking score of −8.34 kcal/mol, forming hydrogen bonds with key residues such as Gln131 and Asn132. The structure–activity relationship (SAR) analysis suggested that halogen substitutions significantly enhanced anticancer activity, particularly chloro and bromo groups at the para positions.

Perike et al. synthesized a series of hybrid molecules containing indole-thiazolidinedione-triazole moieties and evaluated their anticancer activities against four human cancer cell lines: HePG-2 (liver), HCT-116 (colorectal), PC-3 (prostate), and MCF-7 (breast) [81]. The most potent compound, **26** (Figure 5), demonstrated superior anticancer activity, showing IC_50_ values of 4.43 μM against HePG-2, 4.46 μM against HCT-116, 8.03 μM against PC-3, and 3.18 μM against MCF-7, outperforming the standard drug doxorubicin, which exhibited IC_50_ values of 4.46 μM (HePG-2), 5.18 μM (HCT-116), 8.72 μM (PC-3), and 4.13 μM (MCF-7). Molecular docking studies revealed that **26** binds effectively to EGFR, CDK2, and sorcin targets, with a binding affinity of -10.1 kcal/mol against EGFR, forming key interactions with residues like Cys773, Asp776, and Phe771. These findings suggest that compound **26** has the potential to be further developed as a promising anticancer agent due to its potent activity and strong binding affinity to multiple cancer targets.

Parthiban et al. synthesized indole-curcumin derivatives and evaluated their anticancer activities against Hep-2, A549, and HeLa cell lines [82]. The methoxy-substituted indole curcumin derivative (**27**) (Figure 5) showed the most potent anticancer activity, with IC_50_ values of 12 μM against Hep-2, 15 μM against A549, and 4 μM against HeLa cells. These results were comparable to standard drugs doxorubicin (IC_50_ = 10 μM for Hep-2, 0.65 μM for A549, and 1 μM for HeLa) and paclitaxel (IC_50_ = 1.8 μM for Hep-2, 0.175 μM for A549, and 0.08 μM for HeLa). Molecular docking studies revealed that the methoxy-substituted compound **27** strongly binds to GSK-3β, EGFR, and Bcr-Abl proteins, with significant interactions at key active sites. Compound **27** exhibited potent antioxidant activity, reducing DPPH free radicals by 90.50%. These findings suggest that methoxy-substituted indole curcumin derivatives possess dual-action anticancer and antioxidant capabilities, making them promising candidates for further development.

#### 2.1.3. Bcl-2 Inhibitors

Bcl-2 inhibitors have gained significant attention in cancer therapy due to their ability to promote apoptosis in cancer cells by neutralizing the anti-apoptotic functions of the Bcl-2 family proteins [83]. Bcl-2, a key regulator of the mitochondrial pathway of apoptosis, is frequently overexpressed in various cancers, contributing to tumor growth, resistance to chemotherapy, and poor prognosis. Inhibiting Bcl-2 restores the balance between pro-apoptotic and anti-apoptotic signals within the cell, triggering apoptosis and reducing tumor survival [84].

**Figure 6:**
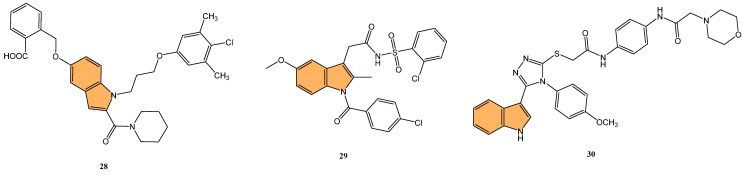
Structures of compounds **28**–**30** as Bcl-2 inhibitors.

Given the therapeutic potential of Bcl-2 inhibitors, Zhang et al. synthesized a series of novel Bcl-2/Mcl-1 dual inhibitors with an indole scaffold and evaluated their potential as anticancer agents [85]. In particular, compound **28** (Figure 6) demonstrated the most potent dual inhibition activity, with sub-micromolar binding affinities for both Bcl-2 and Mcl-1, with Ki values of 0.41 μM and 0.41 μM, respectively, while showing no significant activity against Bcl-XL (IC_50_ > 200 μM). Compound **28** effectively induced apoptosis in HL-60 cells in a dose-dependent manner, with 46.9% apoptosis observed at 80 μM concentration compared to 0.87% in the control. Mechanistic studies revealed that **28** increased PARP cleavage and mitochondrial membrane potential, activating intrinsic apoptotic pathways. Molecular docking studies confirmed that **28** binds to the P2, P3, and P4 pockets of Bcl-2 and Mcl-1, forming key hydrogen bonds and hydrophobic interactions that enhance its inhibitory activity.

Following this line of investigation, Liu et al. in 2022 synthesized a series of novel indole derivatives and evaluated their dual inhibitory activity against Bcl-2 and Mcl-1[86]. The tested compounds have shown potent activity, compound **29** (Figure 6) being the most potent with IC_50_ values of 7.63 μM for Bcl-2 and 1.53 μM for Mcl-1, comparable to the reference compound AT-101 (IC_50_ = 2.60 μM for Bcl-2 and 1.19 μM for Mcl-1). Molecular docking studies revealed that **29** interacts with the active sites of Bcl-2 and Mcl-1 proteins, forming hydrogen bonds and Van der Waals interactions, especially with residues such as Gln96 and Phe101 in Bcl-2. Despite its potent binding affinity, **29** exhibited moderate antiproliferative activity, with an IC_50_ of 69 μM against Jurkat cells, lower than AT-101 (IC_50_ = 3.12 μM). These findings suggest that while **29** is a promising dual inhibitor, further structural optimization is needed to enhance its antitumor efficacy.

In a related effort, Almehdi et al. designed and synthesized a series of novel indole-based Bcl-2 inhibitors to evaluate their anticancer activity against Bcl-2-expressing cancer cell lines [87]. Interestingly, compound **30** (Figure 6) demonstrated the most potent activity, particularly against MCF-7 breast cancer cells, with an IC_50_ of 0.83 μM, as well as against A549 lung cancer cells (IC_50_ = 0.73 μM). For comparison, the standard reference gossypol showed an IC_50_ of 4.43 μM for MCF-7 and 3.45 μM for A549. The compound **30** also demonstrated effective inhibition of Bcl-2 using an ELISA assay, with an IC_50_ of 1.2 μM, which was only two-fold less potent than gossypol (IC_50_ = 0.62 μM). Compound **30** induced significant apoptosis in cancer cells, with a 43-fold increase in early apoptosis and a 111-fold increase in late apoptosis, alongside G1/S phase cell cycle arrest. Molecular docking studies revealed stable binding interactions between **30** and the Bcl-2 protein, involving hydrogen bonding, pi–pi stacking, and hydrophobic interactions with residues such as Arg-60, Phe-63, and Glu-95, indicating strong binding affinity. These results suggest that **30** is a promising candidate for further development as a selective Bcl-2 inhibitor with potent anticancer properties.

#### 2.1.4. Carbonic Anhydrases IX and XII Inhibitors

Carbonic anhydrases (CAs) are a family of zinc-containing enzymes that catalyze the reversible conversion of carbon dioxide and water to bicarbonate and protons, playing a crucial role in pH regulation [88,89]. There are multiple isoforms of CAs, with CA IX and CA XII being particularly important in cancer. These isoforms are often overexpressed in hypoxic tumor environments, where they help maintain intracellular pH homeostasis, aiding cancer cell survival, invasion, and metastasis under acidic conditions [90,91,92]. Given their involvement in tumor growth and progression, developing selective CA inhibitors, particularly against CA IX and CA XII, has emerged as a promising strategy for cancer therapy. Targeting these isoforms can disrupt the pH balance in cancer cells, leading to reduced proliferation, enhanced sensitivity to other therapies, and apoptosis, making CA inhibitors a valuable tool in anticancer drug development [93,94].

**Figure 7:**
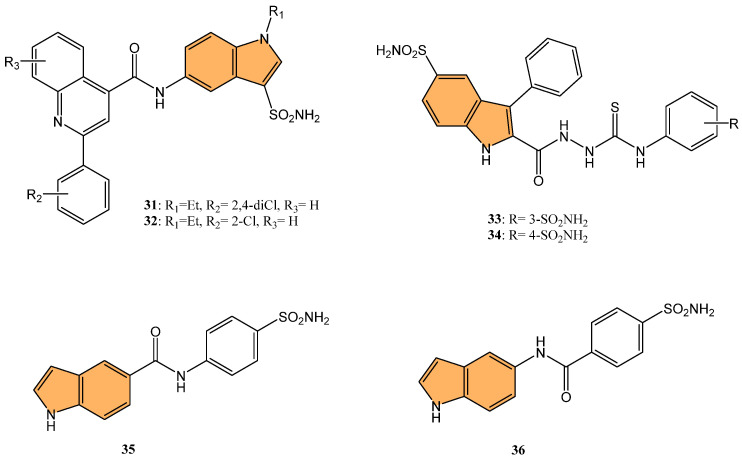
Structures of compounds **31**–**36** as CA inhibitors.

In light of this, Singh et al. synthesized a series of quinoline/pyridine indole-3-sulfonamide hybrids. They evaluated their selective inhibition against carbonic anhydrase (CA) isoforms, particularly the tumor-associated isoforms hCA IX and XII [95]. Results indicated that compounds **31** and **32** (Figure 7) exhibited the most potent inhibition of hCA IX, with Ki values of 1.47 μM and 1.57 μM, respectively. Both compounds showed minimal inhibition against the cytosolic isoforms hCA I and II, demonstrating their selectivity toward tumor-associated isoforms. Compound **32**—with a 2-chloro substitution on the quinoline ring—was particularly effective, forming hydrogen bonds with Thr199 in the active site of hCA IX, as revealed through molecular docking studies. Therefore, compounds **31** and **32** can be considered promising leads for developing selective anticancer agents targeting hCA IX, which plays a significant role in cancer cell survival and metastasis.

Demir-Yazıcı et al. synthesized a series of 2-(hydrazinocarbonyl)-3-phenyl-1H-indole-5-sulfonamide-based thiosemicarbazides and evaluated their potential as selective inhibitors of tumor-associated human carbonic anhydrase (hCA) isoforms IX and XII [96]. Compounds **33** and **34** (Figure 7) demonstrated the most potent inhibition of hCA XII, with Ki values of 0.69 nM and 0.87 nM, respectively, significantly outperforming the standard inhibitor acetazolamide (Ki = 5.7 nM). Both compounds also exhibited strong inhibition of hCA IX, with Ki values of 2.1 nM and 1.4 nM, respectively. Molecular docking and molecular dynamics simulations confirmed favorable binding interactions between these compounds and the active sites of hCA IX and XII, suggesting their potential for further development as selective anticancer agents targeting these isoforms.

Nguyen and co-workers synthesized and evaluated a series of indole-based benzenesulfonamides for their antitumor activity, particularly focusing on their inhibitory effects on carbonic anhydrase IX (CA IX) in hypoxic cancer cells [97]. Among these, **35** and **36** (Figure 7) showed the most potent cytotoxicity against breast cancer cell lines MCF-7 and SK-BR-3, with IC_50_ values close to 50 μM under CoCl2-induced hypoxic conditions. These compounds suppressed the expression of CA IX, which plays a critical role in tumor growth and migration under hypoxic conditions. In CA IX-knockdown cells, the cytotoxic effects of **35** and **36** were significantly diminished, confirming the role of CA IX inhibition in their antitumor activity. Furthermore, **35** and **36** significantly enhanced the anticancer efficacy of doxorubicin (DOX) when combined, particularly in SK-BR-3 cells, where the combination treatment showed strong synergism. The combination also further inhibited cancer cell migration, highlighting the potential of these compounds as adjuvant therapies in breast cancer treatment.

#### 2.1.5. Estrogen Receptor Modulators

Estrogen receptors, particularly estrogen receptor alpha (ERα), play a pivotal role in the progression of hormone-dependent breast cancers. Targeting ERα has become a central approach in developing new therapeutic agents to inhibit estrogen signaling and halt cancer cell proliferation [98]. There has been growing interest in designing novel indole-based compounds in recent years due to their ability to interact with ERα and exhibit potent anticancer properties.

**Figure 8:**
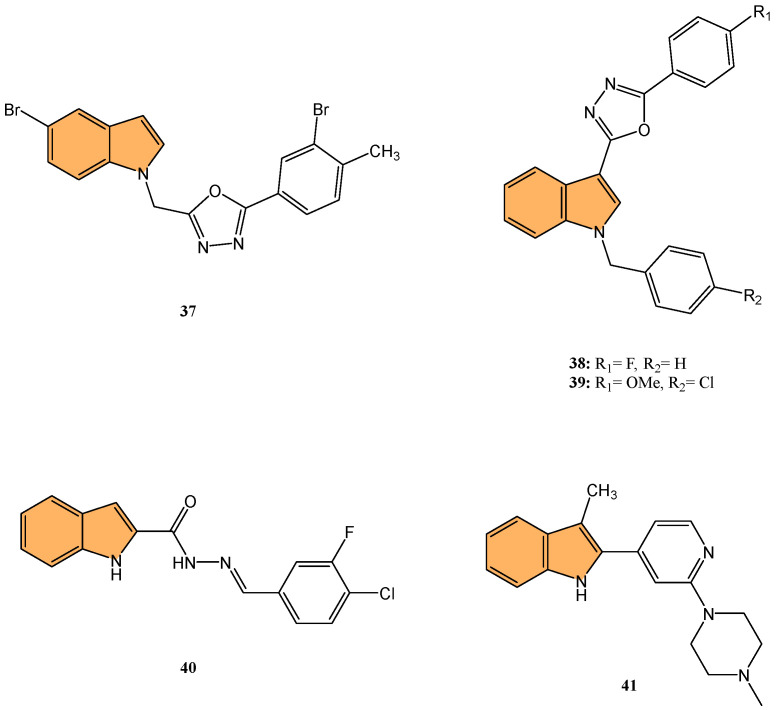
Structures of compounds **37**–**41** as anticancer agents.

For example, Sreenatha et al. designed and synthesized 1,3,4-oxadiazole-indole derivatives and evaluated their anticancer activity, particularly focusing on estrogen receptor alpha (ERα) inhibition [99]. Of these, compound **37** (Figure 8) demonstrated the most potent activity with an IC_50_ of 10.56 μM against MDA-MB-468 cells and 22.61 μM against MDA-MB-231 cells. It also showed an IC_50_ of 5.27 μM in an ERα-specific binding assay, indicating a strong affinity for the estrogen receptor. Computational studies, including density functional theory (DFT) and molecular docking, revealed that **37** forms stable interactions with the active site of ERα, involving key residues like Phe404 through π–π stacking and hydrogen bonds. Additionally, molecular dynamic simulations (MDS) showed that **37** maintains stable binding with ERα throughout a 50 ns simulation, with minimal fluctuations in the protein–ligand complex.

Similarly, Kaur et al. designed and synthesized new indole-oxadiazole derivatives and evaluated their potential to inhibit estrogen receptor alpha (ER-α) for breast cancer treatment [100]. Compounds **38** and **39** (Figure 8) stood out for their potent antiproliferative activity against ER-α-positive T-47D breast cancer cells, with IC_50_ values of 3.24 μM and 1.72 μM, respectively, compared to the standard drug bazedoxifene (IC_50_ = 12.78 μM). Additionally, compound **39** showed a remarkable 1589-fold higher binding affinity for ER-α (213.4 pM) compared to bazedoxifene (339.2 nM), while compound **38** exhibited an IC_50_ of 446.6 nM in the ER-α binding assay. Western blot analysis demonstrated that both compounds effectively reduced ER-α protein levels, with 64.97% and 23.71% reductions, respectively, indicating a suppression of estrogen signaling. Molecular docking studies confirmed that both compounds bind to the active site of ER-α, causing conformational changes that inhibit receptor activity. The pharmacokinetic studies also revealed that both compounds exhibit drug-like properties, making them promising candidates for further development as anti-breast-cancer agents targeting ER-α.

#### 2.1.6. HIF-1α Inhibitors

Hypoxia-inducible factor-1α (HIF-1α) is a transcription factor crucial in cellular adaptation to low oxygen levels (hypoxia), a common feature in the tumor microenvironment. Under hypoxic conditions, HIF-1α stabilizes and translocates to the nucleus, activating the transcription of various genes involved in angiogenesis, metabolism, and survival [101]. This adaptation promotes tumor growth, invasion, and metastasis by enhancing blood vessel formation (angiogenesis) and altering metabolic pathways to support rapid cell proliferation. Targeting HIF-1α in cancer therapy disrupts these adaptive mechanisms, inhibiting tumor progression and improving treatment outcomes [102,103].

Keskin et al. synthesized twelve novel indole-based hydrazone derivatives and evaluated their cytotoxicity against HCT116 colon cancer cells, A549 lung cancer cells, and healthy lung fibroblast cells (MRC-5) [104]. Compound **40** (Figure 8) stood out for its potent anticancer activity, with IC_50_ values of 2.0 μM against HCT116 and 12.5 μM against A549 cells, while displaying moderate toxicity toward healthy cells (IC_50_ = 27.0 μM). Notably, **40** exhibited a high selectivity index (SI = 13.5) for HCT116, indicating strong selectivity for cancer cells over normal cells. Molecular docking studies revealed that **40** binds to the HIF-1α protein, forming critical hydrogen bonds with Asp201 and coordinating with the Fe^2+ ^ ion at the protein’s active site. The interaction of **40** with HIF-1α, a key regulator of tumor adaptation to hypoxic conditions, suggests that it inhibits cancer cell survival by targeting the HIF-1α pathway, making it a promising candidate for selective anticancer therapy.

Rahman et al. synthesized a series of novel 3-methyl indole-based analogs via Pd-catalyzed Suzuki–Miyaura and Buchwald–Hartwig coupling reactions, targeting HIF-1α inhibition [105]. These compounds were evaluated for their anticancer potential against the Mia PaCa-2 pancreatic cancer cell line. Compound **41** (Figure 8) exhibited the most potent antiproliferative activity with an IC_50_ value of 73.63 μM. Molecular docking studies demonstrated that **41** effectively binds to the HIF-1α active site, with a binding affinity of −7.7 kcal/mol, forming hydrogen bonds with key residues such as Asp201, Gln147, and Thr196 as well as hydrophobic interactions with Leu186 and Trp296. The docking data combined with in silico ADMET predictions indicate that **41** holds strong potential for further development as an anti-pancreatic-cancer agent targeting the HIF-1α pathway.

#### 2.1.7. HDAC Inhibitors

**Figure 9:**
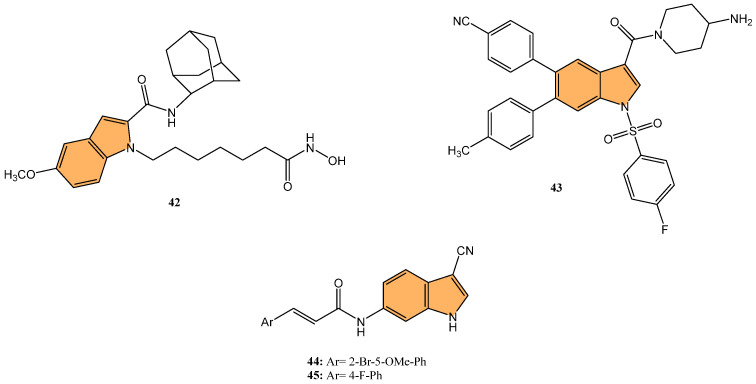
Structures of compounds **42**–**45** as anticancer agents.

Histone deacetylases (HDACs) regulate gene expression by removing acetyl groups from histones, leading to chromatin condensation and transcriptional repression. In cancer, HDACs are often dysregulated, promoting tumor growth and survival [106]. Targeting HDACs with inhibitors can reactivate tumor suppressor genes and induce cancer cell death, offering a promising therapeutic strategy [107]. Jiang et al. synthesized indole-based hydroxamic acid derivatives and evaluated their inhibitory activity against HDACs as potential anticancer agents [108]. The most potent compound, **42** (Figure 9), exhibited strong inhibition of HDAC1 and HDAC6 with IC_50_ values of 1.16 nM and 2.30 nM, respectively, outperforming the standard HDAC inhibitor suberanilohydroxamic acid (SAHA). Compound **42** significantly increased the acetylation of histone H3 in a dose-dependent manner, induced G2/M cell cycle arrest, and promoted apoptosis in HCT116 cancer cells. In addition, it showed potent antiproliferative activity against various cancer cell lines, including A549, MDA-MB-231, SGC7901, HCT116, and HL60, with IC_50_ values as low as 0.02 μM in HL60 cells. In vivo studies in an HCT116 xenograft mouse model further demonstrated that compound **42** inhibited tumor growth by up to 71.79%, with better efficacy and lower toxicity than SAHA, making it a promising candidate for further development as an HDAC inhibitor for cancer therapy.

#### 2.1.8. LSD1 Inhibitors

Lysine-specific demethylase 1 (LSD1), also known as KDM1A, is a key epigenetic regulator involved in the demethylation of histone H3 at lysine 4 (H3K4) and lysine 9 (H3K9), influencing gene expression and chromatin remodeling. LSD1 is critical in various biological processes, including cell differentiation and proliferation [109]. As a result, targeting LSD1 has emerged as a promising strategy to combat cancer by inhibiting its enzymatic activity and restoring normal gene expression patterns [110]. Building on the significance of targeting LSD1 in cancer therapy, Zhang et al. synthesized a series of novel indole derivatives and evaluated their potential as highly potent LSD1 inhibitors [111]. Compound **43** (Figure 9) emerged as the most active LSD1 inhibitor, with an IC_50_ value of 0.050 μM. This compound also demonstrated significant antiproliferative effects against A549 lung cancer cells, with an IC_50_ value of 0.74 μM. In vivo studies showed that **43** had favorable metabolic stability, with a half-life (t1/2) of 6.27 h for oral administration and 8.78 h for intravenous administration, as well as a strong antitumor effect in A549 xenograft models. Additionally, **43** regulated genes are involved in cancer progression, particularly by modulating the PI3K/AKT pathway and upregulating IGFBP3, a key player in cancer cell apoptosis.

#### 2.1.9. IMPDH Inhibitors

Inosine 5′-monophosphate dehydrogenase (IMPDH) is a key enzyme in the de novo synthesis of guanine nucleotides, catalyzing the conversion of inosine monophosphate (IMP) to xanthosine monophosphate (XMP) [112]. This enzyme is crucial for cell proliferation and is often overexpressed in various cancers, including colorectal cancer. Overexpression of IMPDH promotes tumor growth and progression by enhancing nucleotide biosynthesis, which supports rapid cell division. Targeting IMPDH with specific inhibitors can disrupt this pathway, reducing nucleotide availability and inhibiting cancer cell proliferation. IMPDH is a promising therapeutic target in cancer treatment strategies [113].

In expanding on the potential of IMPDH inhibitors in cancer treatment, Jia et al. synthesized a series of novel indole acrylamide derivatives. They evaluated their inhibitory activity against the IMPDH enzyme [114], particularly focusing on the hIMPDH2 isoform, which is highly expressed in tumor cells. Compounds **44** and **45** (Figure 9) showed the most potent inhibition of hIMPDH2, with IC_50_ values of 2.948 μM and 4.207 μM, respectively. These compounds also demonstrated significant cytotoxicity against SW480 human colon cancer cells, with IC_50_ values of 15.31 μM for 14n and 15.34 μM for **45**. Molecular docking studies revealed that both compounds form strong π–π interactions with the purine ring of inosine monophosphate (IMP) and hydrogen bonds with key residues such as Asp274 within the active site of IMPDH, making them promising leads for developing new anticancer agents targeting the IMPDH pathway, particularly for treating colon cancer.

#### 2.1.10. Other Anticancer Mechanisms

Another important target in cancer therapy is the 14-3-3η protein, a member of the 14-3-3 family—a group of regulatory proteins that play a crucial role in signal transduction, cell cycle control, and apoptosis by binding to phosphorylated serine/threonine residues on target proteins. The 14-3-3η isoform, in particular, has been associated with various cancers due to its role in promoting cell survival, proliferation, and metastasis [115]. Overexpression of 14-3-3η has been observed in cancers such as breast, lung, liver, and prostate cancer, where it contributes to oncogenic signaling by stabilizing and activating key cancer-related proteins like AKT, RAF, and BAD [116]. 

**Figure 10:**
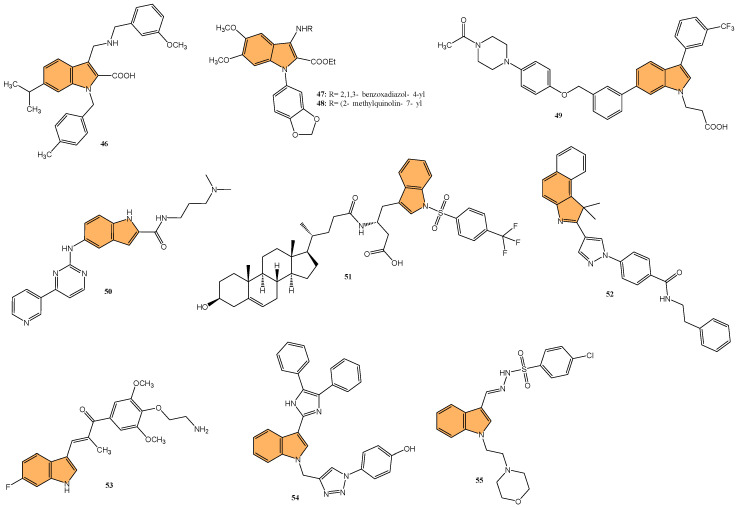
Structures of compounds **46**–**55** as anticancer agents.

In light of the critical role that 14-3-3η plays in cancer progression, targeting this protein has become a promising strategy for therapeutic intervention. Gao et al. synthesized a series of novel 1H-indole-2-carboxylic acid derivatives targeting the 14-3-3η protein [117]. After multiple rounds of optimization, compound **46** (Figure 10) exhibited the best affinity for 14-3-3η and demonstrated potent inhibitory activities against several human liver cancer cell lines, including Bel-7402, SMMC-7721, SNU-387, Hep G2, and Hep 3B cells. Notably, **46** showed superior efficacy against chemotherapy-resistant Bel-7402/5-Fu cells with an IC_50_ of 4.55 μM, compared to sorafenib’s IC_50_ of 13.31 μM. In addition to its antiproliferative effects, **46** induced G1-S phase cell cycle arrest and promoted apoptosis in liver cancer cells, as confirmed by increased cleaved caspase-3 and PARP levels. Molecular docking studies revealed that **46** binds effectively to 14-3-3η, forming hydrogen bonds with key residues, such as Lys50, Arg132, and Tyr133. This suggests that **46** is a promising candidate for HCC treatment, particularly in overcoming chemotherapy resistance.

Yao et al. designed and synthesized a series of 1-benzo [1,3]dioxol-5-yl-3-N-fused heteroaryl indoles and evaluated their antiproliferative activity against various cancer cell lines [118], including prostate (LNCaP), pancreatic (MIA PaCa-2), and acute lymphoblastic leukemia (CCRF-CEM). The most potent compounds, **47** and **48** (Figure 10), had IC_50_ values ranging from 328 to 644 nM against CCRF-CEM and MIA PaCa-2 cells. Mechanistic studies revealed compound **48** induced S-phase cell cycle arrest and apoptosis in CCRF-CEM cells. The structure–activity relationship (SAR) analysis indicated that a 5,6-dimethoxy moiety and a 2-ethyl ester group on the indole scaffold were essential for good antiproliferative potency, highlighting the potential of these derivatives as promising leads for further development as anticancer agents.

Deng et al. synthesized a series of novel Mcl-1 inhibitors bearing an indole carboxylic acid moiety and evaluated their potential for anticancer activity [119]. Compound **49** (Figure 10) displayed the most promising activity with a Ki value of 0.37 μM for Mcl-1 and showed selectivity by not inhibiting Bcl-2 or Bcl-xL. Compound **49** also demonstrated moderate growth inhibition in HL-60 leukemia cells with a GI_50_ value of 3.15 μM, outperforming the control UMI-77 (GI_50_ = 7.78 μM). Mechanistic studies indicated that **49** induces apoptosis in a Mcl-1-dependent manner by disrupting the Mcl-1/Bim protein–protein interaction, as confirmed through co-immunoprecipitation assays. Additionally, **49** exhibited good microsomal and plasma stability, along with favorable pharmacokinetic properties, including a half-life of 4.21 h and an oral bioavailability of 11.10%. In a 4T1 xenograft mouse model, compound **49** significantly inhibited tumor growth, achieving a 52.34% reduction in tumor size at a 40 mg/kg dose.

Qin et al. designed and synthesized a series of 5-((4-(pyridin-3-yl) pyrimidine-2-yl)amino)-1H-indole-2-carboxamide derivatives, targeting the orphan nuclear receptor Nur77 [120], known for its role in inducing apoptosis in cancer cells. Remarkably, compound **50** (Figure 10) exhibited the most potent antiproliferative activity across multiple cancer cell lines, including HepG2, HeLa, and MDA-MB-231, with IC_50_ values of 9.04, 3.75, and 3.24 μM, respectively. Compound **50** demonstrated stronger Nur77-binding activity (KD = 354 nM in SPR assay) than the reference compound celastrol (KD = 292 nM). Further mechanistic studies revealed that **50** induces Nur77-dependent apoptosis by promoting its translocation from the nucleus to mitochondria, triggering mitochondrial membrane potential loss, and enhancing PARP cleavage in HepG2 cells. In vivo, compound **50** significantly inhibited tumor growth in a HepG2 xenograft model with a tumor growth inhibition rate of 71.8% without causing significant toxicity to major organs.

Guidetti et al. synthesized a novel series of 1-(phenylsulfonyl)-1H-indole derivatives targeting the EphA2 receptor, which plays a key role in glioblastoma progression [121]. Results showed that compound **51** (Figure 10) exhibited the most potent antiproliferative activity, with an IC_50_ value of 2.6 μM against EphA2. Compound **51** effectively inhibited the EphA2–ephrin-A1 interaction and suppressed EphA2 phosphorylation in U251 glioblastoma cells at concentrations of 30 μM. Additionally, **51** significantly reduced U251 cell proliferation in vitro, achieving complete inhibition of cell growth at 30 μM after 24 h and inducing cell death after 48 h of treatment. Molecular docking studies confirmed that **51** binds to the EphA2 receptor’s ligand-binding domain (LBD) by occupying a hydrophobic pocket, with the trifluoromethyl group displacing a key water molecule.

Ramle et al. synthesized and evaluated five novel benz[e]indole pyrazolyl-substituted amides for their cytotoxicity against the HCT 116 colorectal carcinoma cell line [122]. Among these, compound **52** (Figure 10) demonstrated the most potent anticancer activity, with an IC_50_ value of 0.17 μg/mL, showing significantly higher efficacy compared to the standard drug cisplatin (IC_50_ = 29.43 μg/mL). Notably, **52** exhibited selective cytotoxicity, showing minimal toxicity toward normal colon fibroblast cells (CCD-18Co), highlighting its potential for selective cancer targeting. Molecular docking studies indicated that the mechanism of action for **52** involves binding to the DNA minor groove, where its benz[e]indole scaffold fits into the groove, facilitating interaction with key nucleotides, making it a potent and selective anticancer agent for colorectal cancer, warranting further exploration in preclinical models.

Du et al. synthesized and optimized a series of fluoro-substituted indole-chalcone derivatives, particularly targeting colorectal cancer (CRC) cells [123]. One standout derivative, compound **53** (Figure 10), featuring an amino-terminus on the 4-methoxyphenyl group, exhibited potent in vitro activity against HCT-116 CRC cells with an IC_50_ in the low nanomolar range. Compound **53** induces G2/M phase arrest through upregulation of cyclin B1 and significantly increases reactive oxygen species (ROS) production, suggesting its mechanism involves cell cycle disruption and oxidative stress. In vivo, **53** demonstrated superior antitumor efficacy, reducing tumor growth by 65.3% and 73.4% at doses of 5 and 10 mg/kg/day, respectively, in an HCT-116 xenograft mouse model, outperforming taxol, which showed a 54.1% inhibition at 7 mg/kg. Importantly, **53** exhibited improved safety and tolerability, with no signs of major organ toxicity, particularly in the brain and colon, which were problematic with previous derivatives. Overall, compound **53** emerges as a promising candidate for CRC chemotherapy, offering superior efficacy and a more favorable safety profile than standard treatments.

Veeranna et al. synthesized a series of 1,2,3-triazole tethered indole derivatives and evaluated their anticancer activity against MCF-7 breast cancer and HepG2 hepatocellular carcinoma cell lines using the MTT assay[124]. The derivative that demonstrated the highest potency was compound **54** (Figure 10), featuring a 4-hydroxy group, with IC_50_ values of 53.17 μM against HepG2 and 72.8 μM against MCF-7 cells, outperforming the standard drug doxorubicin. Molecular docking studies revealed that this compound demonstrated strong binding affinities to Aurora kinase-1 and DNA topoisomerase-2 alpha, with a lower binding energy than doxorubicin.

Gaur et al. synthesized a series of novel indole-based arylsulfonylhydrazides and evaluated their anticancer activity against estrogen receptor-positive (MCF-7) and triple-negative (MDA-MB-468) breast cancer cell lines [125]. Compound **55** (Figure 10), featuring a 4-chlorophenyl substituent, exhibited the most potent anticancer activity, with IC_50_ values of 13.2 μM against MCF-7 cells and 8.2 μM against MDA-MB-468 cells. Compound **55** showed no significant toxicity toward noncancerous HEK-293 cells, suggesting selective cytotoxicity toward cancer cells. The selectivity index (SI) values for compound 5f were 36.6 and 58.9 for MDA-MB-468 and MCF-7 cells, respectively, indicating their strong selectivity. The structure–activity relationship (SAR) analysis revealed that the presence of the 4-chlorophenyl group contributed to the compound’s enhanced activity. Overall, compound **55** shows great potential for further development as a selective breast cancer treatment.

### 2.2. Antimicrobial Activity

#### 2.2.1. Antibacterial Activity

**Figure 11:**
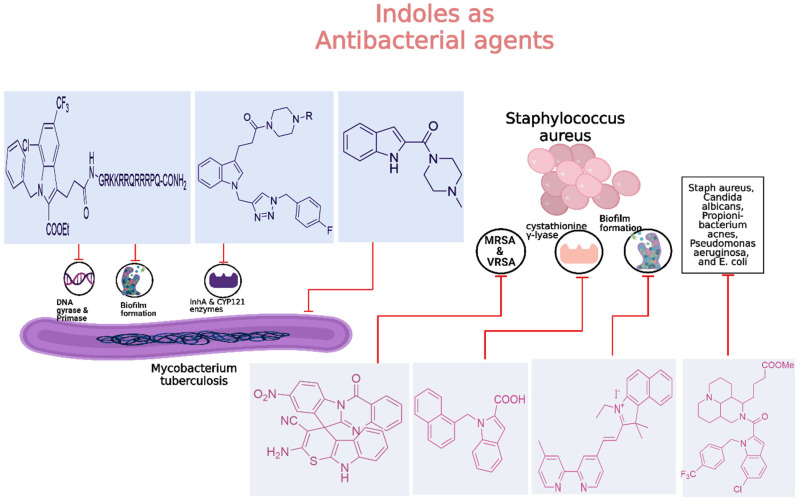
Indole derivatives as antibacterial agents.

Tuberculosis (TB) continues to be a major global health concern, particularly due to the rise in drug-resistant strains [126,127,128]. Developing new anti-tubercular agents is essential to address these challenges and improve treatment efficacy [129]. Indole derivatives exhibit significant potential as antibacterial agents owing to their ability to disrupt bacterial cell walls and inhibit essential bacterial enzymes, which makes them particularly effective against resistant strains (Figure 11).

**Figure 12:**
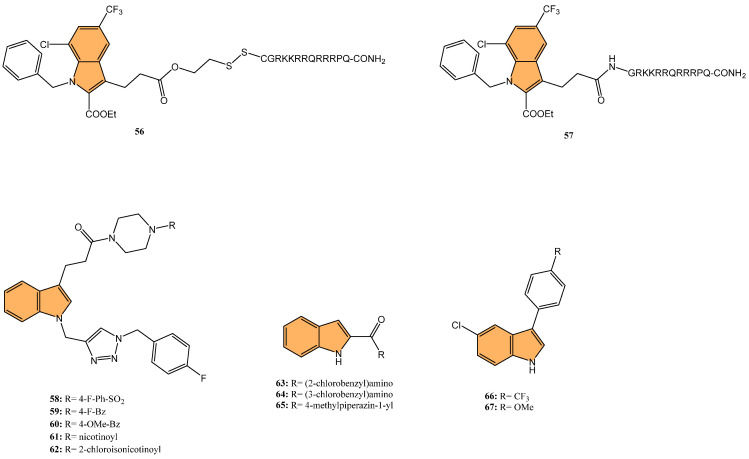
Structures of compounds **56**–**67** as anti-tubercular agents.

Dewangan et al. synthesized a series of cell-penetrating peptide conjugates of indole-3-acetic acid-based DNA primase and gyrase inhibitors to evaluate their anti-tubercular potential [130]. These conjugates were tested against planktonic and biofilm cultures of *Mycobacterium smegmatis*, a model organism for *Mycobacterium tuberculosis*. The conjugates significantly outperformed the free inhibitor molecules, demonstrating more than 50-fold increased activity with minimal inhibitory concentration (MIC) values ranging from 1.9 to 3.9 μM, compared to the inhibitor alone (MIC = 250 μM). The most effective conjugates, **56** and **57** (Figure 12), also impaired biofilm formation, reducing it by more than 99% at MIC levels, surpassing the activity of the standard drug isoniazid. Mechanistic studies showed that these conjugates inhibited DNA primase and gyrase enzymes, with **57** inhibiting gyrase activity at a concentration of 1.85 μM compared to 250 μM for the free inhibitor. These results suggest that the conjugation of cell-penetrating peptides significantly enhances the delivery and efficacy of DNA replication inhibitors, offering a promising therapeutic approach for treating drug-resistant tuberculosis.

Another study was conducted by Reddyrajula et al., where fifty novels 1,2,3-triazole-incorporated indole-piperazine derivatives were evaluated as potential anti-tubercular agents against *Mycobacterium tuberculosis* H37Rv [131]. Five compounds (**58**, **59**, **60**, **61**, and **62**) (Figure 12) exhibited significant anti-tubercular activity with an MIC of 1.6 µg/mL, which is two-fold more potent than the standard drug pyrazinamide and equipotent to isoniazid. The N-1,2,3-triazolyl indole-piperazine derivatives displayed superior activity to simple and N-benzyl indole-piperazine analogs. Furthermore, the active compounds demonstrated selective antibacterial activity, particularly against *Staphylococcus aureus* and *Escherichia coli*, while being non-toxic to VERO cells (IC_50_ > 300 µg/mL). Molecular docking studies revealed strong interactions with the *M. tuberculosis* InhA and CYP121 enzymes, confirming the compounds’ mechanism of action. Additionally, in silico ADME analysis predicted good oral bioavailability, making these derivatives promising for further development as anti-tubercular agents.

In addition to these efforts to combat tuberculosis, Bhakhar et al. synthesized 24 indole-2-carboxamide derivatives as potential anti-tubercular agents and evaluated them against *Mycobacterium tuberculosis* H37Rv [132]. Compounds **63**, **64**, and **65** (Figure 12) exhibited the most promising anti-TB activity, with MIC values of 12.5 μg/mL for **63** and **64** and 3.125 μg/mL for **65**. Compound **65** showed a 32% growth inhibition in RAW 264.7 cells in cytotoxicity assays. Structure–activity relationship (SAR) analysis indicated that piperazine substitutions enhanced anti-tubercular activity, with 4-methyl piperazine (in **65**) demonstrating the highest potency. Molecular docking studies revealed that **65** binds effectively to the mmpL3 protein in *M. tuberculosis*, forming key hydrogen and pi–sigma bonds, and molecular dynamics simulations confirmed the stability of the ligand–protein complex.

Etchart et al. synthesized a series of novel 3-phenyl-1H-indole derivatives and evaluated their antimycobacterial activity against *Mycobacterium tuberculosis*, including drug-resistant strains [133]. Of all the compounds tested, 66 (Figure 12) displayed the greatest potency, with an MIC of 8.4 μM against the H37Rv strain, significantly outperforming other derivatives. Compound **66** also maintained its activity against multidrug-resistant Mtb strains (PT2, PT12, and PT20), with MIC values of 8.4 μM. Additionally, **66** exhibited a time-dependent bactericidal effect in time-kill assays, reducing the bacterial count to undetectable levels at 2x MIC (40 μM) after 21 days. Cytotoxicity studies revealed that **66** inhibited HepG2 and Vero cell viability at concentrations below 30 μM, indicating potential toxicity. However, compound **67** (Figure 12), with an MIC of 19.4 μM, showed no significant cytotoxicity or genotoxicity, making it a promising lead for further development as an anti-tuberculosis agent.

**Figure 13:**
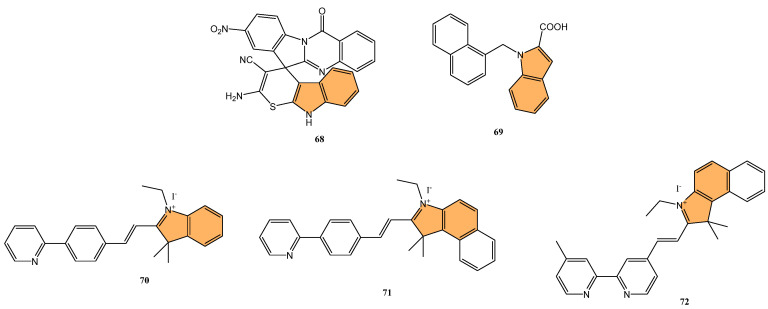
Structures of compounds **68**–**72** as antibacterial agents.

Given the ongoing challenge of antimicrobial resistance, developing new antibiotics targeting drug-resistant bacteria is crucial. Leena et al. developed a series of 24 spirofused tryptanthrin-thiopyrano [2,3-b] indole hybrids using green synthetic methods [134], evaluating their antibacterial activity against methicillin-resistant *Staphylococcus aureus* (MRSA) and vancomycin-resistant *S. aureus* (VRSA). The nitro-substituted compound **68** (Figure 13) exhibited the most potent antibacterial activity, with an MIC of 0.25 μg/mL, comparable to levofloxacin. Compound **68** demonstrated concentration-dependent bactericidal activity, with a post-antibiotic effect (PAE) of 4 hours at 10x MIC, significantly longer than vancomycin’s 2-hour PAE. Additionally, **68** synergized with linezolid compared to individual treatments, reducing bacterial load by 2.1 log10 cfu/mL in combination. Molecular docking studies identified the amino group in compound **68** as a critical binding site. Compound **68** also exhibited good metabolic stability, with a half-life greater than 120 min in rat liver microsomes. It is a promising candidate for further development as an anti-staphylococcal agent.

Kuzovlev et al. synthesized a series of naphthyl-substituted indole and pyrrole carboxylic acids. They evaluated their ability to inhibit bacterial cystathionine γ-lyase (bCSE) [135], an enzyme present in bacterial defense mechanisms. The most potent compound, **69** (Figure 13), exhibited strong inhibitory activity against bCSE with an IC_50_ value significantly lower than the reference inhibitor NL2. Compound **69** showed a 3.6-fold selectivity for bCSE over human CSE (hCSE). In combination with kanamycin, ampicillin, and norfloxacin, **69** potentiated their effectiveness, reducing the MIC of these antibiotics against *Staphylococcus aureus* (including methicillin-resistant *S. aureus*). Additionally, molecular docking and molecular dynamics simulations revealed that **69** binds effectively at a novel active site between bCSE monomers. ADMET studies indicated that **69** had favorable drug-like properties, including high plasma stability, moderate lipophilicity, and low intrinsic clearance.

Zhang et al. synthesized three novel photoactivated indole-pyridine chemotherapeutics designed to combat bacterial infections and biofilms, particularly those formed by *Staphylococcus aureus* [136]. These compounds, **70**, **71**, and **72** (Figure 13), showed potent antimicrobial and antibiofilm activity when activated by light. Under light irradiation, **70** and **72** exhibited MIC_90_ of 0.78 µM against *S. aureus*, comparable to methicillin (MIC_90_ = 1.56 µM), with no significant activity in the dark. These compounds disrupted biofilm formation at concentrations as low as 12.5 µM, and **72** also demonstrated a strong ability to destroy mature *S. aureus* biofilms. Mechanistic studies revealed that **72**, in particular, generated both singlet oxygen and superoxide anions, leading to reactive oxygen species (ROS) production and membrane damage. This dual photoactivated mechanism—targeting both cellular components and biofilms—suggests these compounds have strong potential as photodynamic antimicrobial agents.

**Figure 14:**
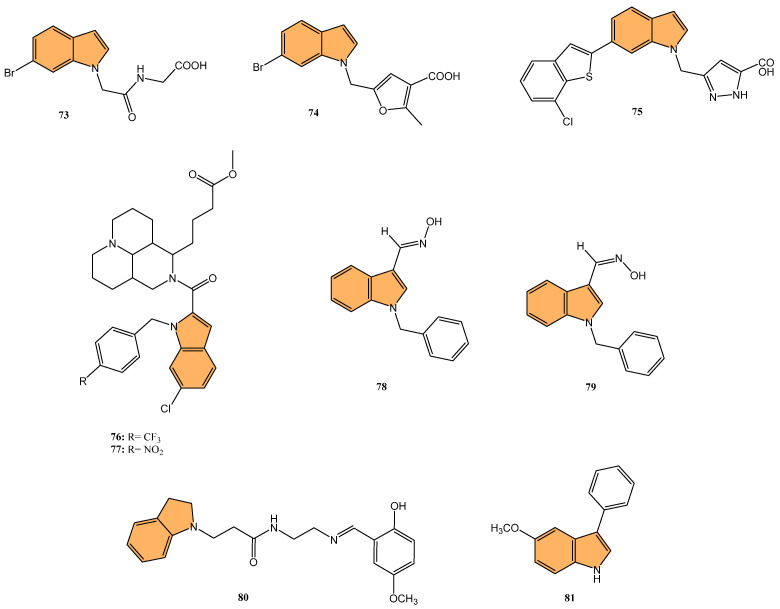
Structures of compounds **74**–**81** as antibacterial and antifungal agents.

Potapov et al. synthesized three indole-based inhibitors (**73**, **74**, and **75**) (Figure 14) targeting bacterial cystathionine γ-lyase (bCSE)[137], an enzyme responsible for hydrogen sulfide (H_2_S) production in pathogenic bacteria like *Staphylococcus aureus* and *Pseudomonas aeruginosa*. These inhibitors enhance the efficacy of antibiotics by reducing bacterial resistance. Among the inhibitors, **74** showed the most promise, inhibiting bCSE with an IC_50_ value of 0.7 μM, while **75** exhibited greater potency but slightly lower selectivity against human CSE. These inhibitors were synthesized using a 6-bromoindole scaffold, with various functional groups attached via Pd-catalyzed cross-coupling reactions. Compound **73**, the simplest series, was synthesized in gram quantities for biological testing. The inhibitors effectively enhanced the antibiotic activity against resistant bacterial strains, positioning them as potential adjuvants in antimicrobial therapies.

Li et al. synthesized 29 novel marine-indole derivatives and evaluated their antibacterial activity against *Staphylococcus aureus,* *Candida albicans*, Propionibacterium acnes, *Pseudomonas aeruginosa*, and *Escherichia coli* [138]. Interestingly, compounds **76** and **77** (Figure 14) exhibited the highest efficacy against *S. aureus*, with MIC values of 0.021 mg/mL and 0.031 mg/mL, respectively. Compound **76** also demonstrated strong activity against *C. albicans* (MIC = 2.806 mg/mL) and *P. acnes* (MIC = 0.030 mg/mL), outperforming standard antibiotics like penicillin G sodium and fluconazole. A 3D-QSAR analysis revealed that steric structure, hydrophobic interactions, and hydrogen bonding were key factors contributing to the antibacterial activity of these compounds. Molecular docking studies highlighted that **76** formed stable complexes with bacterial proteins through hydrogen bonds and hydrophobic interactions, inhibiting their function.

Kalatuwawege et al. synthesized a series of syn- and anti-isomers of N-substituted indole-3-carbaldehyde oxime derivatives to evaluate their urease inhibitory activity against *Helicobacter pylori* [139]. They discovered that compound **78** (Figure 14) demonstrated the most potent inhibition with an IC_50_ of 0.0345 mM. At the same time, compound **79** (Figure 14) also showed strong activity with an IC_50_ of 0.0516 mM, both outperforming the standard thiourea (IC_50_ = 0.2387 mM). Molecular docking studies revealed that compounds **79** and **78** form strong metal–acceptor interactions with Ni^2+^ ions at the active site of urease. The anti-isomer configuration of compound **78** exhibited more favorable interactions than the syn-isomer of compound **79**, correlating with its superior inhibitory activity. The study indicates that N-substituted indole-3-carbaldehyde oximes, particularly compound **78**, hold great potential for the development of novel urease inhibitors.

#### 2.2.2. Antifungal Activity

**Figure 15:**
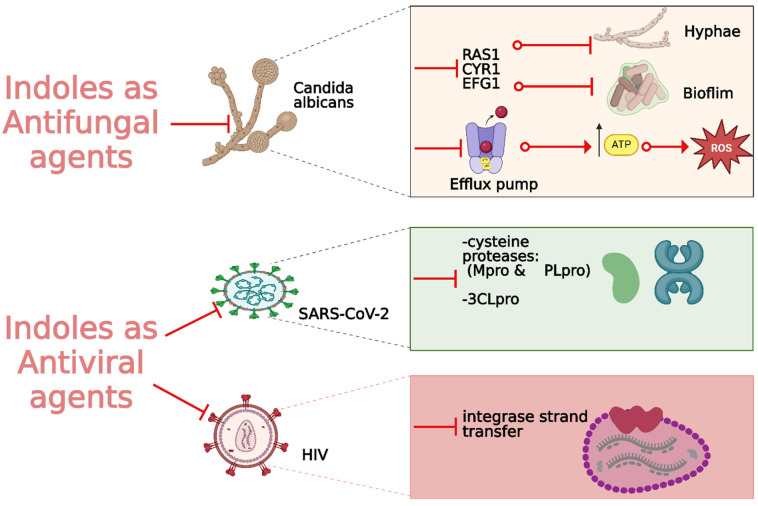
Indole derivatives as antifungal and antiviral agents.

Fungal infections, particularly those caused by drug-resistant strains, pose a significant threat to public health, necessitating the development of new antifungal agents. Indole derivatives have emerged as valuable scaffolds in developing antifungal agents due to their ability to interact with various biological targets in fungi (Figure 15).

Ma et al. synthesized a series of novel indole and indoline derivatives and evaluated their antifungal activity against both fluconazole-sensitive and azole-resistant strains of *Candida albicans* [140]. A particularly notable compound, derivative **80** (Figure 14), demonstrated the most potent antifungal effect, with an IC_50_ of 21 µg/mL against the fluconazole-resistant strain ATCC10231FR, while fluconazole showed no activity (IC_50_ > 200 µg/mL). Compound **80** also exhibited strong antifungal activity against other strains, including the fluconazole-sensitive strain SC5314 (IC_50_ = 22 µg/mL) and various clinical isolates, with consistent inhibition of biofilm formation and hyphal growth. Mechanistic studies revealed that **80** acts through the Ras-cAMP-PKA signaling pathway significantly downregulating key virulence genes such as RAS1, CYR1, and EFG1, which are involved in the growth and development of *C. albicans* hyphae and biofilms. In addition to its potent antifungal activity, **80** showed low cytotoxicity toward human epithelial cells (16HBE), with a CC_50_ greater than 200 µg/mL, suggesting that it could serve as a promising candidate for the treatment of drug-resistant fungal infections without significant toxicity to human cells.

Wu et al. synthesized new indole derivatives and evaluated their antifungal properties against *Candida albicans*, including fluconazole-resistant strains [141]. In particular, 3-phenyl-5-methoxyindole (compound **81**) (Figure 14) emerged as the most potent, with an MIC_80_ value of 126.04 μg/mL against the fluconazole-resistant strain SC5314. Combined with fluconazole, compound **81** displayed strong synergistic effects, reducing the MIC_80_ to 3.25 μg/mL and achieving a fractional inhibitory concentration index (FICI) of 0.03, highlighting its potential to restore fluconazole sensitivity. Mechanistic studies indicated that compound **81** inhibited the yeast-to-hyphae transition, suppressed biofilm formation, and reduced the activity of efflux pumps in *C. albicans*. It also decreased intracellular ATP levels and induced mitochondrial dysfunction by increasing reactive oxygen species (ROS) production. In an in vivo model, compound **81** significantly enhanced the survival rate of larvae infected with *C. albicans*, demonstrating both efficacy and safety. 

#### 2.2.3. Antiviral Activity

**Figure 16:**
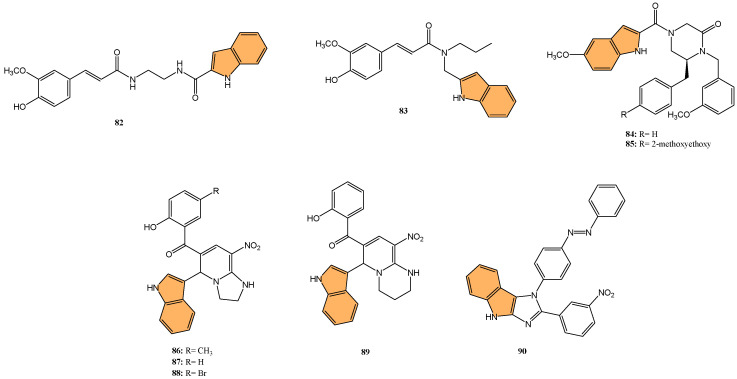
Structures of compounds **82**–**90** as antiviral agents against SARS-CoV-2.

With the global urgency to find new antiviral therapies for SARS-CoV-2, researchers have been exploring different chemical compounds to combat the virus [142]. Indole derivatives have shown promise due to their broad-spectrum antiviral properties, making them candidates for further investigation (Figure 15) [143]. Verzola et al. synthesized indole-based ferulic acid derivatives and evaluated their antiviral activity against SARS-CoV-2 in vitro [144]. It was found that compounds **82** and **83** (Figure 16) demonstrated promising antiviral effects, reducing the number of SARS-CoV-2 genomic copies in a dose-dependent manner, with IC_50_ values of 70.85 µM and 68.28 µM, respectively. These compounds showed no significant cytotoxicity up to 100 µM in uninfected Vero cells, indicating a good safety profile. Interestingly, the antiviral activity of these derivatives was not linked to the inhibition of SARS-CoV-2 cysteine proteases (Mpro and PLpro) or the human cysteine protease cathepsin L. Instead, their mechanism of action may be associated with their antioxidant properties, particularly the phenolic hydroxyl group from the ferulic acid moiety. This suggests that these indole-ferulic acid hybrids may have potential as therapeutic agents against SARS-CoV-2, warranting further investigation into their mechanisms of action and potential development as antiviral drugs.

Soleymani et al. developed QSAR models and performed molecular docking studies on 81 isatin and indole derivatives to evaluate their potential as inhibitors of the SARS-CoV 3CL protease (3CLpro) [145], a key enzyme in the viral replication cycle. The QSAR models were generated using CORAL software and Monte Carlo optimization, leading to the identification of key molecular descriptors influencing inhibitory activity. Compounds **84** and **85** (Figure 16), both containing indole scaffolds, emerged as the most potent inhibitors, with IC_50_ values of 0.043 µM and 0.025 µM, respectively. These compounds exhibited strong binding affinity to the 3CLpro active site with binding energies of −9.6 and −9.7 kcal/mol. Molecular docking confirmed the interactions between these compounds and the key residues in the SARS-CoV 3CLpro active site, including HIS41, CYS145, and GLU166. ADMET analysis further supported their drug-like properties, indicating good human intestinal absorption and low toxicity, making them promising leads for further development as anti-SARS-CoV inhibitors.

Jayabal et al. developed a green and regioselective one-pot method to synthesize 3-substituted indole and 2-substituted pyrrole-based 1,2-dihydropyridine and azaxanthone derivatives [146], aiming to explore their antiviral potential against SARS-CoV-2 and the Delta Plus K417N variant. Molecular docking studies revealed that five synthesized compounds exhibited higher binding affinity for the SARS-CoV-2 main protease (Mpro) than the reference drug remdesivir. Among these, compound **86** (Figure 16) demonstrated the most potent inhibitory effect, with a docking score of −8.6 kcal/mol, indicating a strong interaction with the viral protease. Compounds **87**, **88**, and **89** (Figure 16) showed promising binding affinities for the spike glycoprotein of the Delta Plus variant, with docking scores that surpassed remdesivir’s binding performance.

Geedkar et al. synthesized a series of azo-anchored 3,4-dihydroimidazo [4,5-b]indole derivatives using an ultrasonic-assisted method. They evaluated their potential inhibitory activity against the main protease (Mpro) of SARS-CoV-2 [147] through molecular docking studies. From the synthesized series, compound **90** (Figure 16) demonstrated the highest binding affinity to Mpro, with a MolDock score of −168.76, outperforming standard drugs like remdesivir (MolDock score: −168.56) and Paxlovid (MolDock score: −158.51). The binding interactions of compound **90** with the active site involved hydrogen bonds with key residues, including CYS145 and HIS164, which play a critical role in the protease’s catalytic function. Cytotoxicity tests on HEK293 cells revealed that **90** had minimal toxicity, with cell survival rates exceeding 89% at concentrations up to 100 μM. The study outcomes highlight that compound **90**, along with other synthesized derivatives, has strong potential as a therapeutic candidate for COVID-19, paving the way for further in vitro and in vivo evaluations.

**Figure 17:**
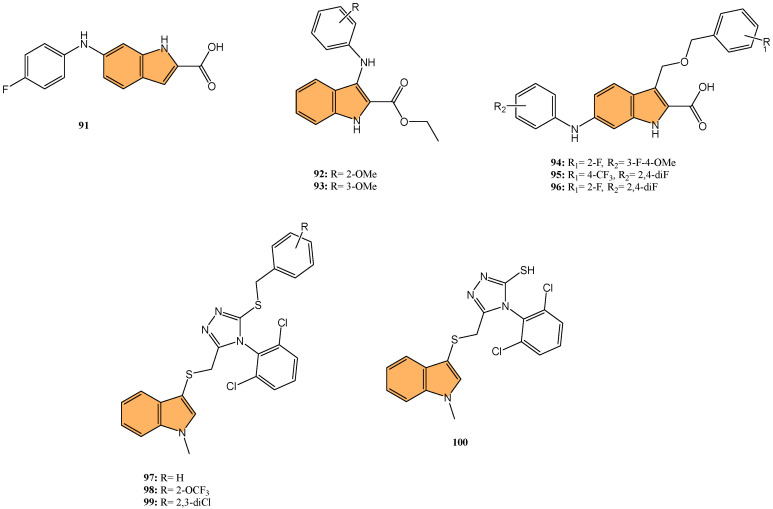
Structures of compounds **91**–**100** as antiviral agents.

Continuing the exploration of indole-based compounds as antiviral agents, Zhang et al. designed and synthesized a series of indole-2-carboxylic acid derivatives as novel HIV-1 integrase strand transfer inhibitors (INSTIs) [148]. Through structural optimization, compound **91** (Figure 17) emerged as the most potent inhibitor, displaying an IC_50_ value of 3.11 μM, significantly improving over the parent compound’s IC_50_ of 32.37 μM. Compound **91** achieved its high potency by forming strong metal–chelation interactions with two Mg^2+^ ions in the integrase active site and establishing π–π stacking interactions with viral DNA, crucial for inhibiting the integrase strand transfer process. Other derivatives, such as 92 and 93 (Figure 17), also demonstrated improved inhibitory activities, with IC_50_ values around 10 μM. These compounds showed low cytotoxicity in human cells (CC_50_ > 80 μM), indicating a favorable safety profile. Molecular docking studies further confirmed the strong binding affinities of these compounds to the integrase active site, making them promising candidates for developing new HIV-1 integrase inhibitors with the potential to combat drug-resistant strains.

Similarly, in a focused effort to develop more potent HIV-1 integrase inhibitors, Wang and coworkers synthesized a series of indole-2-carboxylic acid derivatives as novel HIV-1 INSTIs [149] through virtual screening and structural optimization. Of particular interest, compound **94** (Figure 17) exhibited the most potent inhibitory activity with an IC_50_ value of 0.13 µM, significantly outperforming the parent compound (IC_50_ = 12.41 µM) and approaching the potency of the positive control raltegravir (IC_50_ = 0.06 µM). Compound **94** demonstrated strong chelation with two Mg^2+^ ions at the integrase active site and formed key π–π stacking interactions with viral DNA. Other derivatives, such as **95** and **96** (Figure 17), also displayed improved activity, with IC_50_ values of 0.39 µM and 0.64 µM, respectively. These compounds exhibited low cytotoxicity toward human MT-4 cells (CC_50_ > 80 µM), indicating a favorable therapeutic index.

Extending the search for novel antiviral agents beyond coronaviruses and HIV, Ji et al. synthesized novel indole-containing triazole derivatives and evaluated their antiviral activity against influenza A virus (IAV) [150]. In this investigation, compound **97** (Figure 17) stood out as the most potent compound exhibiting anti-IAV activity, with an IC_50_ value of 1.34 μM, outperforming the control drug ribavirin (IC_50_ = 27.76 μM). Compound **97** also demonstrated low cytotoxicity (CC_50_ > 100 μM) and a high selectivity index (SI > 74.63), indicating a favorable safety profile. Other derivatives, such as **98** and **99** (Figure 17), also showed significant antiviral activity, with IC_50_ values of 1.41 μM and 2.14 μM, respectively. Structural variations, such as different benzyl substituents, were found to influence antiviral potency. Compound **100** (Figure 17), an intermediate without aromatic substitution, also displayed strong anti-IAV activity (IC_50_ = 1.48 μM), suggesting that simple aliphatic chain substitutions on the sulfur atom are promising for further optimization.

### 2.3. Anti-Inflammatory Activity

**Figure 18:**
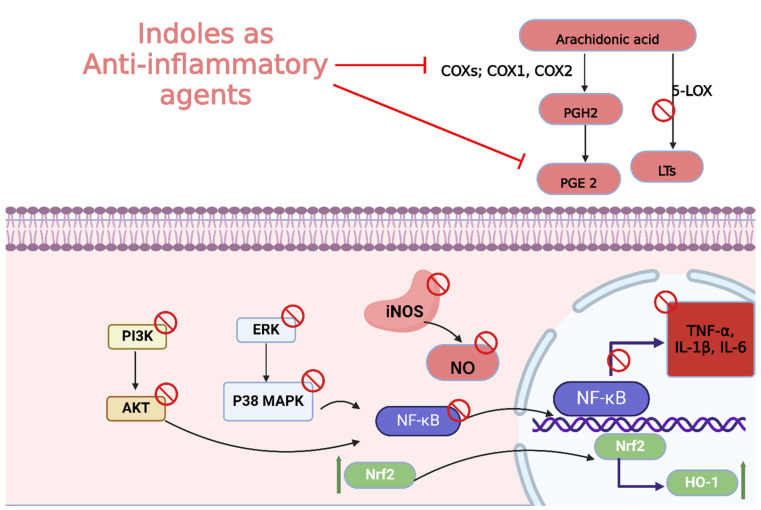
Different mechanisms of indole derivatives with anti-inflammatory activities.

Indole derivatives have shown considerable potential as anti-inflammatory agents due to their ability to modulate various inflammatory pathways. These compounds have been extensively explored for their therapeutic potential in treating inflammatory diseases by inhibiting key enzymes and signaling molecules involved in inflammation (Figure 18) [151].

**Figure 19:**
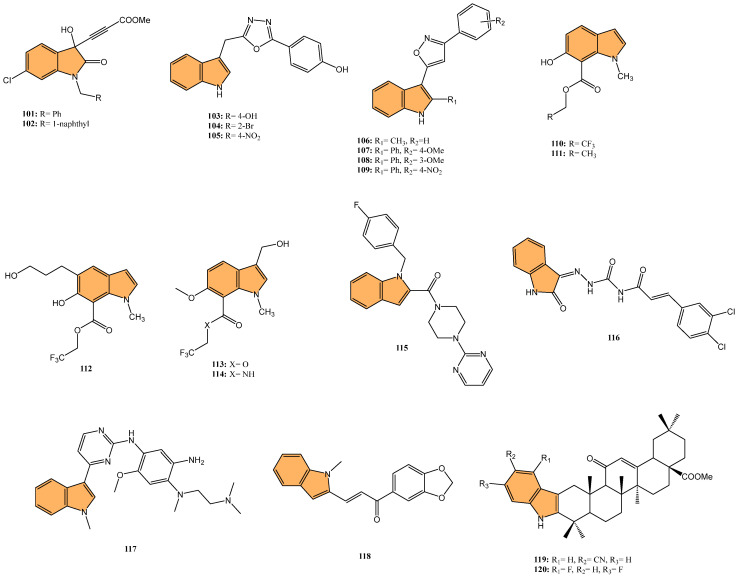
Structures of compounds **101**–**120** as anti-inflammatory agents.

In one such study, Akhtar et al. synthesized a series of indole-derived γ-hydroxy propiolate esters and evaluated their anti-inflammatory activity in vitro and in vivo [152]. Showing the highest inhibition of nitric oxide (NO) production, compound **101** (Figure 19) achieved a remarkable inhibition rate of 101.2% in LPS-stimulated RAW 264.7 cells at a concentration of 10 μM. Additionally, **101** showed significant PGE2 inhibition, reducing its production by 94.52% at 5 μM. In vivo, L-37 demonstrated a dose-dependent anti-inflammatory effect in a xylene-induced ear edema model, achieving an inhibition rate of 85.43% at 100 mg/kg, comparable to the standard drug celecoxib (93.23%). Furthermore, compounds **101** and **102** (Figure 19) significantly downregulated COX-2 expression, with inhibition rates of 38.79% and 42.58%, respectively, and L-39 also inhibited 5-LOX enzyme expression by 71.19% at 5 μM.

Building upon these findings, Kumar et al. synthesized a series of indole-functionalized pyrazoles and oxadiazoles. They evaluated their anti-inflammatory and analgesic properties in vivo [126] as well as their antioxidant potential. In the carrageenan-induced rat paw edema model, compound **103** (Figure 19) exhibited the most potent anti-inflammatory activity, with a 74.07% edema reduction, close to that of indomethacin (92.59%). Analgesic activity was evaluated using the tail-flick method, and compound **103** also demonstrated significant analgesic effects, increasing the reaction time in rats. Compound **104** (Figure 19) showed potent inhibition of COX-2 (63.23%) and moderate selectivity for COX-2 over COX-1 (a selectivity index of 1.49). Antioxidant assays revealed that compounds **104**, **105** (Figure 19), and **103** were the most effective, with IC_50_ values of 1.55, 2.48, and 2.51 μg/mL, respectively, comparable to ascorbic acid (1.18 μg/mL). Molecular docking studies supported the strong binding of these compounds to the COX-2 enzyme.

Similarly, Bhatia et al. synthesized 15 indole-3-substituted isoxazole derivatives and evaluated their anti-inflammatory and analgesic activities [153]. With the strongest anti-inflammatory activity, compounds **106**, **107**, and **108** (Figure 19) reduced carrageenan-induced paw edema by 77.42%, 67.74%, and 61.29%, respectively, comparable to the reference drug indomethacin (83.87%). COX inhibition studies revealed that these compounds displayed 2–3-fold selectivity for COX-2 over COX-1. Compound **106**, in particular, showed the highest COX-2 inhibition (57.45%) and was identified as the lead compound due to its balanced anti-inflammatory, analgesic, and COX selectivity profile. Molecular docking studies confirmed strong binding interactions of the active compounds with the COX-2 catalytic site. Additionally, in vitro antioxidant assays demonstrated that several compounds, including **106** and **109** (Figure 19), exhibited potent free radical scavenging activity, further highlighting their therapeutic potential as anti-inflammatory agents.

In a related study, Faura et al. synthesized indole-based derivatives with tunable functionalities and evaluated their potential as cyclooxygenase-1 (COX-1) inhibitors and anticancer agents [154]. Compounds **110** and **112** (Figure 19) exhibited the most potent COX-1 inhibition, with IC_50_ values of 12.6 µM and 5.6 µM, respectively, while showing high selectivity over COX-2 (selectivity indices >30). In anticancer activity assays, compound **113** (Figure 19) demonstrated the strongest cytotoxic effects, particularly against Hep-G2 liver cancer cells, with an IC_50_ of 7.63 µM, and also showed activity against MCF-7 and LnCaP cells. Molecular docking studies revealed that compounds **110**, **111** (Figure 19), and **112** fit well within the COX-1 binding pocket, forming crucial hydrogen bonds with active site residues such as Tyr-355 and Arg-120. In contrast, **113** and **114** (Figure 19), which were more active against COX-2, showed strong interactions with key residues in the COX-2 binding site. Additionally, compounds like **113** induced cell cycle arrest at the G1/G0 stage and promoted apoptosis through caspase-3 activation, downregulation of Bcl-2, and upregulation of Bax, suggesting a dual role of these compounds in both COX inhibition and cancer cell apoptosis.

Furthering the exploration of indole derivatives, Wang et al. synthesized a series of novel indole and indazole-piperazine pyrimidine derivatives. They evaluated their anti-inflammatory and neuroprotective activities for potential ischemic stroke treatment [155]. Impressively, compound **115** (Figure 19) demonstrated the most potent cytoprotective effects against oxygen-glucose deprivation/reoxygenation (OGD/R)-induced damage in BV2 cells, with cell viability of 72.17%, 83.78%, and 89.74% at concentrations of 0.1, 1, and 10 μM, respectively, outperforming the control edaravone. Additionally, **115** significantly reduced the release of inflammatory mediators, including TNF-α, IL-1β, IL-6, nitric oxide (NO), and prostaglandin E2 (PGE2) from lipopolysaccharide (LPS)-induced BV2 cells. It also exhibited dual inhibition of cyclooxygenase-2 (COX-2, IC50 = 92.54 nM) and 5-lipoxygenase (5-LOX, IC50 = 41.86 nM), making it a strong anti-inflammatory agent. In a middle cerebral artery occlusion (MCAO) rat model, **115** reduced infarct volumes and improved neurological deficit scores, suggesting significant neuroprotective effects.

Targeting acute lung injury (ALI), Zheng et al. designed and synthesized 40 diimide-indole derivatives and evaluated their potential for treating ALI by targeting the NF-κB signaling pathway [156]. With its significant anti-inflammatory properties, compound **116** (Figure 19) achieved an IC_50_ value of 1.05 μM for inhibiting IL-6 production in LPS-stimulated J774A.1 macrophages. In vivo studies showed that compound **116** significantly reduced lung inflammation, decreasing neutrophil infiltration and the wet/dry lung weight ratio in a mouse model of LPS-induced ALI. Mechanistic studies revealed that **116** inhibited NF-κB signaling by blocking the phosphorylation of P65 and preventing its nuclear translocation, thus reducing the expression of inflammatory cytokines such as IL-6 and TNF-α. Additionally, **116** displayed no significant toxicity in mice in acute toxicity tests at 1000 mg/kg doses.

In another investigation, Chen et al. designed and synthesized a series of 4-indole-2-arylaminopyrimidine derivatives and evaluated their anti-inflammatory activity, focusing on ALI [157]. The most potent anti-inflammatory response was observed with compound **117** (Figure 19), which inhibited IL-6 and IL-8 release by 62% and 72%, respectively, in LPS-induced HBE cells. Additionally, compound **117** reduced inflammatory cell infiltration in the lung tissues of mice with LPS-induced ALI and significantly decreased the lung wet/dry ratio, indicating a reduction in pulmonary edema. In vivo, **117** inhibited the release of key inflammatory cytokines, such as IL-1β, IL-6, and TNF-α, and reduced inducible nitric oxide synthase (iNOS) expression. Mechanistic studies revealed that **117** exerts its anti-inflammatory effects by inhibiting the phosphorylation of p38 and ERK in the MAPK signaling pathway, making it a promising candidate for treating ALI and related inflammatory diseases.

Baramaki et al. developed a series of indole-chalcone hybrids and evaluated their analgesic and anti-inflammatory activities in vivo [158]. The chalcones were tested for their antinociceptive effects in hot-plate, tail immersion, and acetic-acid-induced writhing tests at 10 mg/kg. All compounds extended the response latency to thermal stimuli, with most compounds showing significant effects in the tail immersion test. Compound **118** (Figure 19), 1-(1,3-benzodioxol-5-yl)-3-(1-methyl-1H-indol-2-yl)prop-2-en-1-one, stood out for providing the highest efficacy in both analgesia and inflammation models, reducing writhing by 61.74%, which exceeded the standard drug, diclofenac potassium (54.98%). Anti-inflammatory activity was assessed using the carrageenan-induced mouse paw edema model, where compound **118** exhibited significant inhibition of edema. In silico ADME analysis predicted that compound **118** possessed favorable oral bioavailability and drug-like properties, making it a promising candidate for further development as a pain and inflammation treatment.

Jin et al. synthesized a series of oleanolic acid (OA) derivatives containing indole moieties and evaluated their anti-inflammatory activity through in vitro and in vivo studies [159]. Compounds **119** and **120** (Figure 19) demonstrated superior anti-inflammatory activity, showing significantly higher inhibition of nitric oxide (NO) production in LPS-stimulated BV2 cells than OA, with inhibition rates of 43.8% and 54.8% at 8 μM, respectively, compared to OA’s 22.7%. These compounds also exhibited low cytotoxicity, with MTT assays confirming their safety at 16 μM. They also significantly reduced ear swelling in a TPA-induced ear edema model, outperforming both OA and dexamethasone (DXM). Mechanistic studies revealed that compounds **119** and **120** inhibited the expression of pro-inflammatory cytokines (IL-1β, IL-6, and TNF-α) and upregulated anti-inflammatory cytokine IL-10, likely through the inhibition of the NF-κB, MAPK, and PI3K/Akt signaling pathways, while activating the Nrf2/HO-1 pathway.

### 2.4. Antidiabetic Activity

**Figure 20:**
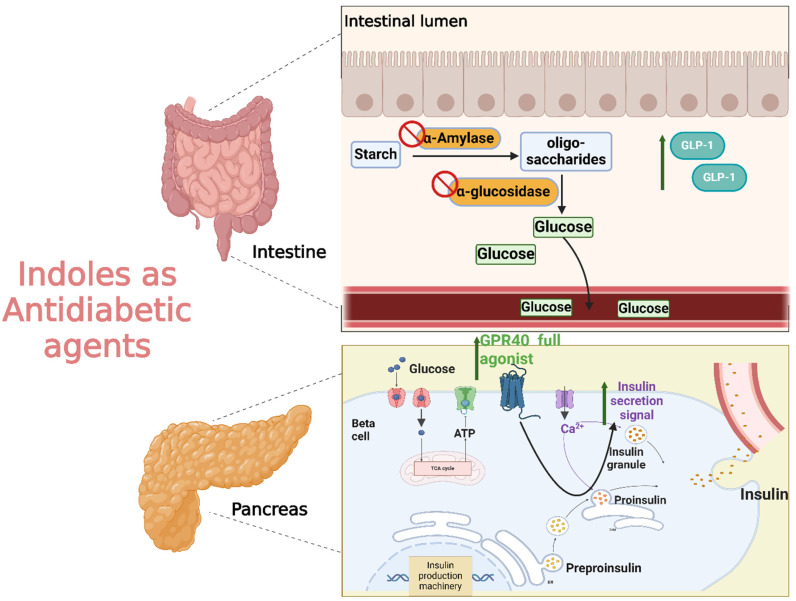
Different mechanisms of indole derivatives with antidiabetic activity.

Indole derivatives have demonstrated significant potential as antidiabetic agents, particularly due to their ability to inhibit key enzymes and regulate pathways involved in glucose metabolism. These compounds are being explored for their effectiveness in managing diabetes mellitus by targeting α-glucosidase, α-amylase, and other metabolic enzymes, which are crucial for controlling blood glucose levels (Figure 20) [160].

**Figure 21:**
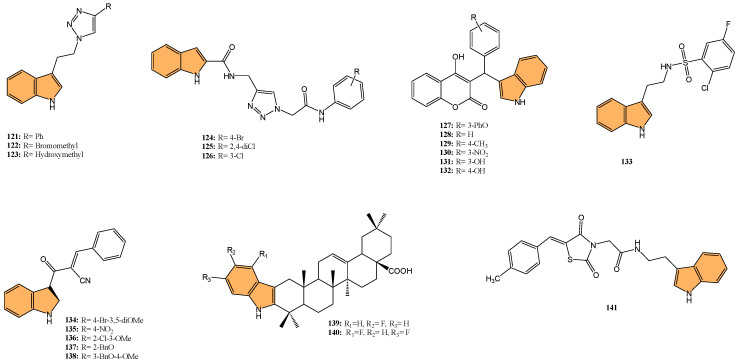
Structures of compounds **121**–**141** as antidiabetic agents.

Ritu et al. synthesized triazole-clubbed indole derivatives and evaluated their antidiabetic potential as α-glucosidase inhibitors [161]. The highest inhibitory activity was observed with compound **121** (Figure 21), which demonstrated an IC_50_ value of 10.1 μM, compared to acarbose’s IC_50_ of 13.5 μM. Compounds **122** and **123** (Figure 21) also showed significant inhibitory activity with IC_50_ values of 12.95 μM and 11.35 μM, respectively. In vivo, studies revealed that these compounds improved body weight and reduced blood glucose levels in diabetic mice. Additionally, the lipid profiles of treated mice showed reduced levels of total cholesterol, triglycerides, and LDL, along with an increase in HDL levels, when compared to the standard drug. Molecular docking studies further supported these results, with **121** showing the highest docking score (−6.73 kcal/mol), indicating strong binding interactions with α-glucosidase’s active site residues, such as PHE166 and GLU271. This suggests that compound **121** holds promise as a potent antidiabetic agent targeting the α-glucosidase enzyme.

Sayahi et al. synthesized a novel series of *N*-phenylacetamido-1,2,3-triazolyl-indole-2-carboxamide derivatives and evaluated their inhibitory activity against α-glucosidase [162]. Compound **124** (Figure 21) demonstrated superior inhibitory activity, recording an IC_50_ value of 26.8 μM, a 28-fold improvement over acarbose (IC_50_ = 752.0 μM). Compound **125** (Figure 21) was the second most potent, with an IC_50_ of 39.6 μM. Structure–activity relationship (SAR) studies indicated that halogen substitutions, especially the 4-bromo group in **124**, significantly enhanced inhibitory effects, while 3-chloro derivatives like **126** (Figure 21) showed the weakest activity with an IC_50_ of 311.3 μM. Kinetic analysis revealed that **124** acted as a competitive inhibitor, with a Ki value of 26.0 μM. Molecular docking confirmed the strong binding of **124** to the active site of α-glucosidase, with interactions involving key residues such as Thr301 and His279.

Similarly, Niri et al. synthesized a series of coumarin-indole hybrids and evaluated their potential as α-glucosidase inhibitors for treating type 2 diabetes [163]. Astonishingly, compound **127** (Figure 21), a 3-phenoxyphenyl derivative, exhibited the most potent inhibitory activity with an IC_50_ value of 116.0 µM, significantly outperforming the standard drug acarbose (IC_50_ = 750.0 µM). Other notable compounds included **128** (IC_50_ = 118.0 µM), **129** (IC_50_ = 167.5 µM), and **130** (IC_50_ = 180.5 µM) (Figure 21). Structure–activity relationship (SAR) studies revealed that phenoxy substitution at the 3-position of the phenyl ring contributed to enhanced inhibitory activity. In contrast, hydroxyl substitution at the same position, as seen in compounds **131** and **132** (Figure 21), resulted in a loss of activity. Kinetic studies showed that **127** is a competitive inhibitor of α-glucosidase with a Ki value of 148 µM. Docking studies confirmed strong binding interactions between **127** and key residues in the α-glucosidase active site, further supporting its potential as a lead compound for developing new antidiabetic agents.

Taha et al. synthesized indole sulfonamide derivatives and evaluated them as α-glucosidase inhibitors [164]. The most potent compound, **133** (Figure 21), exhibited an IC_50_ value of 1.60 µM, showing significantly better inhibitory activity than the standard drug acarbose (IC_50_ = 42.45 µM). Compound **133** was further tested for antidiabetic activity in streptozotocin (STZ)-induced diabetic rats. At a dose of 50 mg/kg, compound **133** reduced blood glucose levels by 24% within 4 h. Long-term studies revealed a 53% reduction in fasting blood glucose levels over 28 days, significantly outperforming the standard drug glibenclamide, which achieved a 41% reduction. Molecular docking studies confirmed compound **133** exhibited strong interactions with key residues of the α-glucosidase active site, such as Arg312 and Asn241, through hydrophobic interactions and hydrogen bonding. These data indicate that indole sulfonamide derivatives have potential as effective antidiabetic agents.

Solangi et al. synthesized indole acrylonitrile derivatives and evaluated them as potential α-glucosidase inhibitors for treating diabetes mellitus [165]. Compound **134** (Figure 21) exhibited the highest inhibitory activity, with an IC_50_ value of 0.53 μM, significantly outperforming the standard drug acarbose (IC_50_ = 2.91 μM). Other active compounds, such as **135**, **136**, **137**, and **138** (Figure 21), also demonstrated strong inhibitory effects, with IC_50_ values ranging from 0.88 to 1.36 μM. Molecular docking studies revealed that compound **134** formed strong hydrophobic interactions and π–π stacking with key residues in the α-glucosidase active site, including Phe157, Phe177, and Phe300, further explaining its potent inhibitory activity.

Wu et al. synthesized a series of oleanolic acid (OA) derivatives infused with indole moieties and evaluated their potential as α-glucosidase inhibitors [166]. The indole-OA derivatives exhibited superior α-glucosidase inhibitory activity compared to OA and OA methyl ester derivatives, with IC_50_ values ranging from 4.02 to 5.30 μM, significantly better than OA itself (IC_50_ = 5.52 μM). Compounds **139** and **140** (Figure 21) were identified as the most potent inhibitors. Mechanistic studies revealed that these compounds act as mixed-type α-glucosidase inhibitors by forming a stable ligand–enzyme complex, as demonstrated through biochemical assays, circular dichroism, and molecular docking studies. This highlights the promising nature of indole-OA derivatives as candidates for managing type 2 diabetes mellitus due to their potent inhibitory activity and ability to interfere with glucose metabolism pathways.

Hu et al. synthesized indole derivatives containing thiazolidine-2,4-dione and evaluated their potential as α-glucosidase inhibitors with antidiabetic activity [167]. The most potent inhibitor, compound **141** (Figure 21), recorded an IC_50_ value of 2.35 μM, far more effective than acarbose (IC_50_ = 575.02 μM). Kinetic studies revealed that **141** acts as a mixed-type inhibitor, binding to the enzyme and the enzyme–substrate complex. In vivo antidiabetic studies in diabetic mice showed that oral administration of **141** (50 mg/kg and 100 mg/kg) significantly reduced fasting blood glucose levels and improved glucose tolerance; additionally, **141** ameliorated dyslipidemia by reducing serum cholesterol (TC) and triglycerides (TG) levels. Molecular docking studies indicated that **141** formed strong hydrogen bonds with key residues such as Arg312 and Glu350 in the α-glucosidase active site, contributing to its potent inhibitory effect.

**Figure 22:**
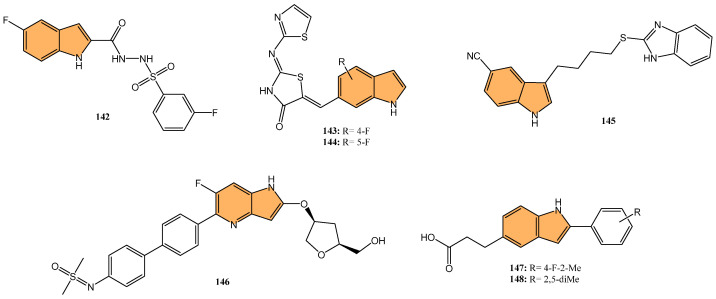
Structures of compounds **142**–**148** as antidiabetic agents.

Taha et al. synthesized indole derivatives and evaluated their potential as inhibitors of α-glucosidase and α-amylase enzymes for managing type 2 diabetes [168]. Achieving the strongest inhibitory effect, compound **142** (Figure 22) exhibited IC_50_ values of 3.80 μM for α-amylase and 3.20 μM for α-glucosidase, outperforming the standard drug acarbose, which showed IC_50_ values of 12.20 μM and 11.20 μM, respectively. Kinetic studies revealed compound **142** acted as a competitive inhibitor of α-amylase and a non-competitive inhibitor of α-glucosidase. Molecular docking studies confirmed strong interactions between compound **142** and key residues in the active sites of both enzymes. The study also demonstrated that compound **142** remained stable in the enzyme binding sites during molecular dynamics simulations, supporting its potential as a therapeutic agent for diabetes.

Khan et al. synthesized thiazolidinone-based indole derivatives and evaluated their inhibitory activities against α-amylase and α-glucosidase enzymes [143]. The most potent compounds, **143** and **144** (Figure 22), exhibited IC_50_ values of 1.80 μM and 1.50 μM for α-amylase, and 2.70 μM and 2.40 μM for α-glucosidase, respectively, significantly outperforming the standard drug acarbose. Structure–activity relationship (SAR) analysis revealed that fluoro substituents on the phenyl ring contributed to enhanced activity, particularly when positioned at the ortho- or meta-positions. Molecular docking studies supported these results, showing strong interactions between the active compounds and the enzyme binding sites, including key residues like Arg312 and Glu350 in α-glucosidase. These findings highlight the promising potential of thiazolidinone-based indole derivatives, especially compounds **143** and **144**, encouraging the audience about the progress in antidiabetic drug development.

Jagadeesan et al. synthesized indole-3-heterocyclic derivatives and evaluated their antidiabetic and antioxidant activities [169]. Compound **145** (Figure 22) showed the most potent α-amylase inhibitory activity with an IC_50_ value of 6.44 μg/mL, compared to the standard drug acarbose (IC_50_ = 5.7 μg/mL). Additionally, compound **145** demonstrated significant antioxidant activity in both DPPH and ABTS assays, with IC_50_ values of 15.62 μg/mL and 12.00 μg/mL, respectively. Molecular docking studies confirmed strong binding interactions between compound **145** and the active site of the α-amylase enzyme, particularly with key residues such as Glu233 and Tyr62.

Exploring alternative mechanisms for diabetes treatment, Tamura et al. synthesized a series of novel indole derivatives. They evaluated them as potent AMP-activated protein kinase (AMPK) activators for treating type 2 diabetes [170]. With the most potent activity, compound **146** (Figure 22) achieved an EC_50_ value in the single-digit nanomolar range for β2-AMPK isoform activation. In vivo studies in diabetic KKAy mice showed that compound **146** led to a dose-dependent improvement in glycemic control, significantly reducing blood glucose and HbA1c levels and decreasing hepatic lipid accumulation. Compound **146** demonstrated strong AMPK activation in skeletal muscle and the liver, significantly enhancing insulin sensitivity. With favorable pharmacokinetic properties, including low clearance, good metabolic stability, and no inhibition of major CYP450 enzymes, compound **146** offers a promising approach for managing metabolic disorders such as type 2 diabetes by targeting AMPK activation, effectively improving glycemic control.

Investigating G-protein-coupled receptor 40 (GPR40) as a novel therapeutic target for type 2 diabetes, Zhao et al. synthesized a series of 2-(disubstituted phenyl)-indole-5-propanoic acid derivatives. They evaluated their potential as full agonists of GPR40 [171]. Compounds **147** and **148** (Figure 22) were identified as the most potent, with EC_50_ values of 40 nM and 417 nM, respectively, demonstrating robust activation of both Gq and Gs signaling pathways. In vitro assays revealed that these compounds significantly enhanced glucose-stimulated insulin secretion (GSIS) and stimulated glucagon-like peptide-1 (GLP-1) release from pancreatic β-cells and enteroendocrine cells, crucial for maintaining glucose homeostasis. In vivo studies further confirmed their efficacy, with compound **148** showing notable glucose-lowering effects in C57BL/6J and db/db mouse models, effectively reducing blood glucose levels and increasing plasma-active GLP-1. Compound **148** exhibited favorable pharmacokinetic properties and improved glycemic control, positioning it as a strong therapeutic candidate for type 2 diabetes by leveraging insulinotropic and incretin-based mechanisms.

### 2.5. In the Management of Neurodegenerative Diseases

#### 2.5.1. Cholinesterase Inhibitors

Indole derivatives have shown significant potential as cholinesterase inhibitors, making them promising candidates for the treatment of neurodegenerative diseases such as Alzheimer’s disease. These compounds target key enzymes such as acetylcholinesterase (AChE) and butyrylcholinesterase (BuChE), which play crucial roles in the pathogenesis of neurodegenerative disorders. Researchers have explored various structural modifications of indole derivatives to develop potent inhibitors with favorable drug-like properties.

**Figure 23:**
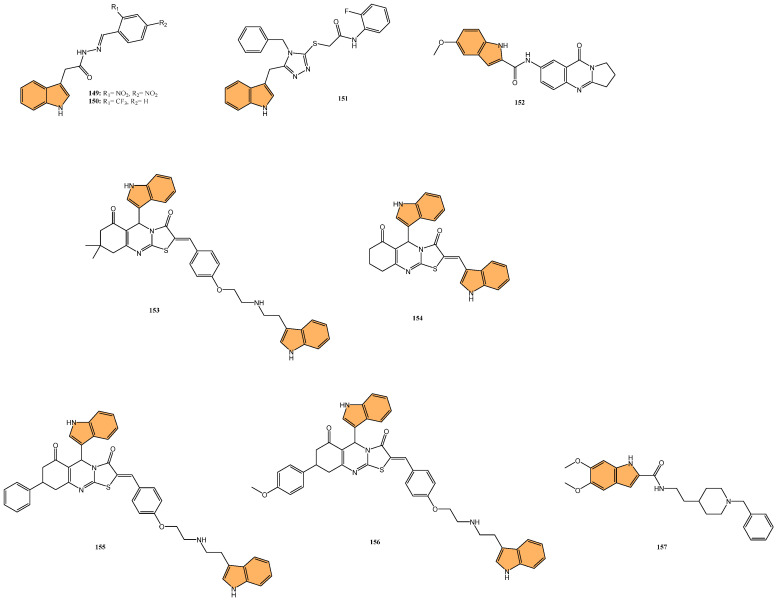
Structures of compounds **149**–**157** as cholinesterase inhibitors.

For instance, Coşar et al. synthesized novel indole-based hydrazide-hydrazone derivatives. They evaluated them for their AChE and BuChE inhibitory activities, targeting potential treatments for Alzheimer’s disease [172]. Compounds **149** and **150** (Figure 23) showed the highest AChE inhibitory activities, with IC_50_ values of 11.33 μM and 26.22 μM, respectively. At the same time, **150** exhibited strong BuChE inhibition with an IC_50_ value of 4.33 μM, closely comparable to the reference drug Galantamine (IC_50_ = 1.26 μM). Molecular docking studies revealed that these compounds formed significant interactions with key residues in the active sites of both enzymes, particularly involving hydrogen bonds and hydrophobic interactions. In silico ADME studies confirmed the drug-likeness of these compounds, making them promising candidates for further development as anticholinesterase agents.

Alım et al. investigated the potential of indole derivatives as dual inhibitors of AChE and BChE [173]. The study revealed compound **151** (Figure 23) was the most potent inhibitor, with IC_50_ values of 0.340 µM for AChE and 1.940 µM for BChE. The reference compound tacrine, a known cholinesterase inhibitor, exhibited IC_50_ values of 57.9 nM for AChE and 3.19 nM for BChE. Molecular docking and dynamics simulations supported these findings, with compound **151** displaying strong binding affinities to both enzymes, with docking scores of −12.240 kcal/mol for AChE and −12.925 kcal/mol for BChE. Compound **151** also formed key interactions with amino acids in the active sites of both enzymes, such as hydrogen bonding with ASP72 and TYR121 in AChE and GLY116 and HIS438 in BChE. This compound demonstrates considerable potential as a therapeutic candidate for Alzheimer’s disease, based on its dual inhibition of both enzymes.

Nerella et al. designed and synthesized a series of deoxyvasicinone-indole hybrids as multifunctional agents targeting Alzheimer’s disease [174]. These compounds were evaluated for their inhibitory effects on AChE and BuChE and their ability to inhibit amyloid-beta (Aβ1-42) aggregation, a hallmark of Alzheimer’s pathology. Compound **152** (Figure 23), in particular, was the most potent inhibitor, with IC_50_ values of 0.12 µM for AChE and 0.15 µM for BuChE. Furthermore, **152** demonstrated significant inhibition of self-induced Aβ1-42 aggregation with an IC_50_ value of 1.21 µM and inhibited AChE-induced Aβ1-42 aggregation by 80.05%. Kinetic analysis revealed compound **152** acts as a mixed-type inhibitor for both enzymes, while molecular docking studies confirmed its strong interaction with the enzyme’s active sites. Given its potent dual inhibitory activity and ability to disrupt amyloid aggregation, compound **152** represents a strong candidate for further development in Alzheimer’s disease therapy.

Nadeem et al. synthesized and evaluated indole core-containing 2-arylidine derivatives of thiazolopyrimidine as multitarget inhibitors for cholinesterases AChE and BChE as well as monoamine oxidase isoforms (MAO-A and MAO-B) [175], aiming to treat Alzheimer’s disease. Compound **153** (Figure 23) showed the highest potency against AChE with an IC_50_ value of 0.042 µM and inhibited BChE with an IC_50_ of 0.63 µM. In addition, compounds **154, 155,** and **156** (Figure 23) emerged as the most potent MAO-B inhibitors with IC_50_ values of 0.13 µM, 0.10 µM, and 0.14 µM, respectively. Kinetic studies showed compound **153** acts as a mixed-type inhibitor, confirmed by a Ki value of 12 nM. The compounds were shown to be non-neurotoxic in MTT assays and could cross the blood–brain barrier, as confirmed by PAMPA-BBB assays. Docking studies supported the experimental data, showing strong interactions with the active sites of the target enzymes, including π–π stacking and hydrogen-bonding interactions with key amino acids.

Banoo et al. designed and synthesized a series of indole-piperidine amides as dual inhibitors targeting cholinesterases (AChE and BChE) and β-secretase (BACE-1) [176], addressing the multifactorial pathology of Alzheimer’s disease. The lead compound, **157** (Figure 23), displayed potent inhibition, with IC_50_ values of 0.32 μM for human AChE and 0.39 μM for human BACE-1, making it the most effective among the series. Kinetic analysis revealed that **157** functions as a mixed-type inhibitor for both enzymes, with Ki values of 0.26 μM for AChE and 0.46 μM for BACE-1, indicating strong binding affinity. Additionally, compound **157** demonstrated significant central nervous system permeability, as evidenced by the PAMPA-BBB assay, making it a promising candidate for further investigation. Molecular docking studies provided insights into the binding mechanisms, showing key interactions between **157** and critical residues in the active sites of AChE and BACE-1, including hydrogen bonding with Asp 32 and π–π stacking with Trp 286. Given the aforementioned promising results, compound **157** could serve as a potential multi-target therapeutic agent for Alzheimer’s disease.

**Figure 24:**
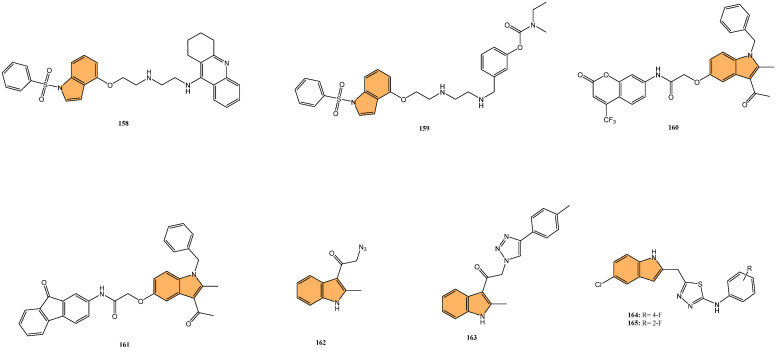
Structures of compounds **158**–**165** as cholinesterase inhibitors.

Wichur et al. designed a series of 1-(phenylsulfonyl)-1H-indole-based multifunctional ligands targeting cholinesterases and 5-HT6 receptors with anti-aggregation properties against amyloid-beta (Aβ) and tau [177], both of which are critical in Alzheimer’s disease progression. Impressively, compound **158** (Figure 24), a tacrine derivative, was found to be a reversible inhibitor of AChE and BChE, with IC_50_ values of 8 nM and 24 nM, respectively, and a Ki value of 13 nM for the 5-HT6 receptor. Additionally, compound **159** (Figure 24), containing a rivastigmine-derived phenyl N-ethyl-N-methylcarbamate fragment, showed selective inhibition of BChE (IC_50_ = 455 nM) and potent anti-tau-aggregation effects, inhibiting tau aggregation by 79%. Both compounds also inhibited amyloid-beta aggregation in vitro (75% for compound **158** and 68% for compound **159** at 10 µM). These compounds exhibited favorable ADMET profiles, with acceptable metabolic stability and no significant cytotoxicity, making them strong candidates for further optimization as potential treatments for Alzheimer’s disease.

Neshat et al. synthesized and evaluated a series of indole-based heterocyclic conjugates as cholinesterase inhibitors for potential Alzheimer’s disease treatment [178]. The study identified compound **160** (Figure 24) (containing a 4-trifluorocoumarin moiety) as the most potent, displaying IC_50_ values of 0.30 µM against AChE and 10.16 µM against BuChE. Another promising compound, **161** (Figure 24) (containing a 9-fluorenone moiety), exhibited IC_50_ values of 0.58 µM for AChE and 15.13 µM for BuChE. Molecular docking studies revealed that **160** and **161** had better binding affinities for AChE (docking scores of −9.06 kcal/mol and −9.03 kcal/mol, respectively) compared to the reference drug donepezil (−8.52 kcal/mol). Additionally, molecular dynamics simulations confirmed stable binding interactions for both compounds, with MMGBSA binding free energy values of −33.10 kcal/mol for 14c and −36.64 kcal/mol for 14h. Both compounds also demonstrated good ADME properties and showed no cytotoxicity against HEK-293 and SH-SY5Y cell lines.

Cetin et al. synthesized and evaluated a series of 3-substituted 2-methyl indole analogs as potential inhibitors of AChE and glutathione S-transferase (GST), which are enzymes linked to Alzheimer’s disease and detoxification processes [179]. Compound **162** (Figure 24) was discovered as the most potent inhibitor, exhibiting IC_50_ values of 0.55 µM for AChE and 1.58 µM for GST. Another potent compound, **163** (Figure 24), showed IC_50_ values of 0.63 µM for AChE and 0.60 µM for GST. The Ki values for these compounds ranged from 0.75 µM to 1.18 µM for AChE and from 0.79 µM to 1.48 µM for GST, indicating strong binding affinity. Molecular docking studies confirmed the strong interactions of these analogs with key residues in the active sites of both AChE and GST, with binding energies ranging from −9.3 to −6.0 kcal/mol for AChE and from −11.1 to −7.5 kcal/mol for GST. These indole derivatives, particularly **162** and **163**, offer potential as multifunctional agents for treating Alzheimer’s disease and other related conditions.

Khan et al. synthesized a series of indole-based thiadiazole derivatives and evaluated them as dual inhibitors of AChE and BuChE for potentially treating Alzheimer’s disease [180]. One particular derivative, compound **164** (Figure 24), demonstrated the most potent inhibitory activity, with IC_50_ values of 0.15 µM for AChE and 0.20 µM for BuChE. Additionally, compound **165** (Figure 24) also showed significant activity with IC_50_ values of 0.35 µM for AChE and 0.50 µM for BuChE. These compounds outperformed the reference drug Donepezil, which had IC_50_ values of 0.21 µM for AChE and 0.30 µM for BuChE. Molecular docking studies confirmed the binding interactions of these compounds with the active sites of AChE and BuChE, including key hydrogen bonding and π–π stacking interactions, particularly involving fluorine-substituted analogs, which enhanced their inhibitory profiles. With their superior activity compared to Donepezil, compounds **164** and **165** show great potential as candidates for further development in Alzheimer’s disease therapies.

#### 2.5.2. Other Mechanisms

**Figure 25:**
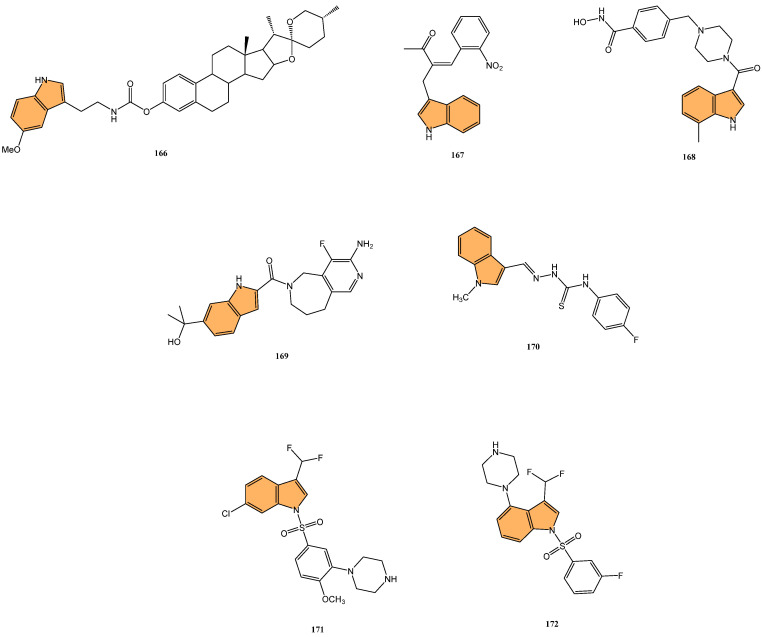
Structures of compounds **166**–**172** targeting neurodegenerative diseases.

Zhou et al. synthesized diosgenin-indole derivatives as dual-functional agents for Alzheimer’s disease treatment [181]. Of these, compound **166** (Figure 25) emerged as the most potent, displaying neuroprotective activities against oxidative stress and neurotoxicity induced by H_2_O_2_ (52.9%), 6-OHDA (38.4%), and beta-amyloid (Aβ1-42) (54.4%). Molecular docking studies confirmed a strong binding affinity of **166** to Aβ1-42, with a binding energy of −40.59 kcal/mol. Compound **166** exhibited favorable blood–brain barrier permeability, with a predicted brain/blood partition coefficient (QPlogBB) of −0.733 and a polar surface area of 85.118 Å², indicating its potential for central nervous system absorption. In vivo, compound **166** significantly improved memory and learning impairments in Aβ-injected mice, making it a promising candidate for Alzheimer’s therapy.

Chiu et al. explored the therapeutic effects of indole derivatives, particularly **167** (Figure 25), in reducing inflammation and oxidative stress in neurotoxin-induced cell and mouse models of Parkinson’s disease [182]. In vitro, **167** alleviated MPP+-induced cytotoxicity in human microglial HMC3 cells and significantly reduced the production of nitric oxide (NO), IL-1β, IL-6, and TNF-α, key markers of inflammation. Furthermore, **167** downregulated the activation of the NLRP3 inflammasome pathway, contributing to its anti-inflammatory properties. In vivo, **167** improved motor functions in MPTP-induced Parkinson’s mice, restored dopamine levels in the striatum, and decreased oxidative stress and neuroinflammation by reducing the activation of microglia and astrocytes. The neuroprotective effects were achieved by upregulating antioxidative enzymes such as SOD2, NRF2, and NQO1, highlighting the potential of **167** as a therapeutic agent for Parkinson’s disease.

Liang et al. synthesized a series of indole-piperazine derivatives as selective histone deacetylase 6 (HDAC6) inhibitors, aiming to treat neurodegenerative diseases like Alzheimer’s disease [183]. Interestingly, compound **168** (Figure 25) emerged as the most potent, exhibiting an IC_50_ of 13.6 nM against HDAC6, with 102-fold selectivity over HDAC1 (IC_50_ = 1390 nM). In vitro, **168** promoted neurite outgrowth in PC12 cells—increasing the percentage of neurite-bearing cells by 34.7% at 3 µM concentration—and demonstrated neuroprotective activity against H_2_O_2_-induced oxidative damage. Compound **168** improved PC12 cell viability up to 78.5% at 10 µM in oxidative stress models. Molecular docking studies revealed that **168** forms stable interactions with the HDAC6 active site, involving π–π stacking with Phe620 and Phe680 and hydrogen bonding with His499.

Nishikawa-Shimono et al. designed and synthesized novel indole derivatives as potent and selective inhibitors of proMMP-9 activation, targeting neurodegenerative diseases such as fragile X syndrome [184]. Utilizing high-throughput screening and structure-based drug design, compound **169** (Figure 25) emerged as the most potent inhibitor, with an IC_50_ value of 44 nM for proMMP-9, showing significant selectivity over proMMP-2 and proMMP-13 (IC_50_ > 30,000 nM for both). Compound **169** also demonstrated high aqueous solubility (>38 μg/mL) and acceptable brain penetration. X-ray crystallography revealed that compound **169** forms hydrogen bonds with Arg106 at the proMMP-9 activation site, contributing to its high inhibitory potency. With its potent inhibitory activity and favorable drug-like properties, compound 169 represents a strong candidate for treating diseases linked to aberrant MMP-9 activity.

Pasha et al. synthesized a series of indole-based thiosemicarbazones and evaluated them as prolyl oligopeptidase (POP) inhibitors for treating neurodegenerative diseases such as Alzheimer’s and Parkinson’s disease [185]. It was found that compound **170** (Figure 25), featuring a 4-fluorophenyl moiety, exhibited the highest inhibitory activity with an IC_50_ value of 5.74 µM. Kinetic studies of **170** revealed concentration-dependent inhibition with a Ki value of 4.31 µM. Molecular docking analysis demonstrated that **170** forms key hydrogen bonds with the active site residues of POP, including Ser554 and Trp595, alongside hydrophobic interactions with Phe173 and Tyr473, contributing to its strong inhibitory activity. The combination of potent inhibition and favorable molecular interactions suggests that compound **170** holds considerable potential as a basis for developing POP inhibitors for neurodegenerative disease therapies.

Yi et al. designed and synthesized a series of novel difluoromethyl-containing 1-((4-methoxy-3-(piperazin-1-yl)phenyl)sulfonyl)-1H-indole derivatives, targeting 5-HT6 receptors for the treatment of Alzheimer’s disease [186]. These compounds were developed by hybridizing two well-known 5-HT6R antagonists, idalopirdine and SB-271046, to improve potency and pharmacokinetic properties. The most potent compound, **171** (Figure 25), exhibited a Ki value of 0.085 nM for 5-HT6R, representing a 10-fold improvement over idalopirdine (Ki = 0.83 nM). In vivo, **171** significantly reversed scopolamine-induced memory deficits in the novel object recognition test, demonstrating its potential as a cognition-enhancing agent. Additionally, **171** showed favorable pharmacokinetic characteristics, including high oral bioavailability (60.9%) and stability in human liver microsomes, which are crucial for its potential as an orally administered drug. The strong receptor affinity and pharmacokinetic properties suggest that **171** is a promising candidate for further development in treating Alzheimer’s disease.

In continuation of their research, Yi et al. further developed a series of difluoromethylated 1-(phenylsulfonyl)-4-(piperazin-1-yl)-1H-indole derivatives [187], focusing on improving the pharmacokinetic and ADME properties of these 5-HT6 receptor antagonists. Among the newly synthesized compounds, **172** (Figure 25) was the most promising, with a Ki value of 0.52 nM for 5-HT6R. Within in vivo cognition-enhancing studies, **172** effectively reversed scopolamine-induced memory deficits in rats, further showing synergistic effects when combined with donepezil, a common Alzheimer’s treatment, by significantly increasing acetylcholine levels in the hippocampus. In addition to its efficacy, **172** demonstrated excellent blood–brain barrier penetration and favorable oral bioavailability, indicating its potential for clinical use as an orally administered drug. Taken together, these data highlight **172** as a potent and selective 5-HT6R antagonist with significant therapeutic potential for Alzheimer’s disease, warranting further investigation.

### 2.6. Antihypertensive Activity

**Figure 26:**
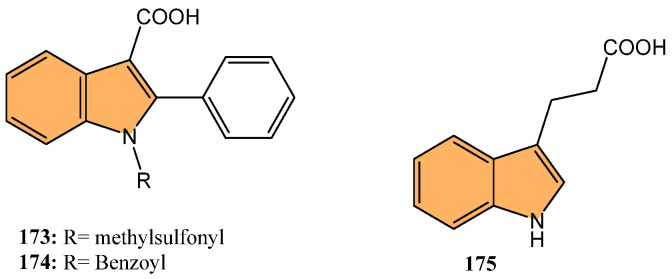
Structures of compounds **173**–**175** with antihypertensive properties.

Danilenko et al. synthesized and evaluated novel indole-3-carboxylic acid derivatives as potential antihypertensive agents, specifically targeting the angiotensin II receptor (AT1R) [188]. These compounds were designed to improve upon known angiotensin II receptor blockers (ARBs) like losartan. Compounds **173** and **174** (Figure 26) were found to exhibit the highest affinity for AT1R, with IC_50_ values of 11.3 nM and 12.3 nM, respectively, comparable to losartan’s IC_50_ of 14.6 nM. In vivo, studies in spontaneously hypertensive rats (SHRs) revealed that **173** and **174** significantly reduced blood pressure, with a maximum decrease of 46.3 mmHg and 48.6 mmHg, respectively, outperforming losartan (42.5 mmHg). The antihypertensive effects of these compounds were sustained for 24 h. Pharmacokinetic studies of compound **174** demonstrated favorable properties, including a half-life of 19.9 h and significant plasma concentration levels, making it a strong candidate for further development as an antihypertensive drug.

Baranwal et al. investigated the effects of indole propionic acid **(175)** (Figure 26) on immune modulation and blood pressure regulation in mice with salt-sensitive hypertension (LSHTN) [189]. The study revealed that mice with LSHTN exhibited decreased serum and fecal IPA levels, increased renal pro-inflammatory T helper 17 (Th17) cells, and decreased anti-inflammatory T regulatory (Treg) cells. Dietary supplementation with IPA significantly lowered systolic blood pressure (SBP) in LSHTN mice, improved sodium handling, and decreased renal Th17 cells while increasing Treg cells. In vitro, IPA directly reduced Th17 cell polarization and increased Treg cell polarization, demonstrating its role in attenuating inflammation and improving blood pressure regulation.

## 3. Future Perspectives

The future of indole derivatives in drug discovery is poised to advance significantly by integrating cutting-edge synthetic methodologies and a deeper understanding of molecular mechanisms. Developing novel indole-based compounds should prioritize the design of multi-targeted agents capable of simultaneously modulating several key biological pathways, a particularly relevant strategy in treating complex diseases such as cancer and neurodegenerative disorders. Leveraging structure-based drug design (SBDD) and quantitative structure–activity relationship (QSAR) models will guide the rational design of indole derivatives with enhanced specificity, reduced toxicity, and optimal pharmacokinetic properties. Furthermore, exploring indole derivatives as epigenetic modulators, kinase inhibitors, and immune checkpoint regulators represents a promising frontier in oncology, where resistance to current therapies remains a significant challenge. The application of advanced drug delivery systems, including nanoparticle-based formulations, could also improve indole-based drugs’ bioavailability and therapeutic index, making them more effective in clinical settings. Collaborative efforts combining medicinal chemistry, computational biology, and clinical research will be critical in translating the potential of indole derivatives into tangible therapeutic benefits.

## 4. Conclusions

Thanks to their wide range of biological activities and significant therapeutic potential, indole derivatives have become a cornerstone in medicinal chemistry. Recent advancements in understanding how these compounds work have highlighted their promise in tackling pressing medical challenges, particularly in areas like cancer, infectious diseases, and inflammatory disorders. For instance, indole hybrids containing a trimethoxy phenyl moiety—resembling colchicine and combretastatin A4—show anticancer activity by inhibiting tubulin polymerization, while indoles with a sulfonamide group inhibit carbonic anhydrase isoform IX, also contributing to anticancer effects. In antimicrobial research, conjugating indoles with cell-penetrating peptides enhances delivery and bioavailability, providing a novel strategy to combat bacterial infections. Hybridization with oleanolic acid has yielded indole derivatives exhibiting both antidiabetic and anti-inflammatory activities, while benzyl indoles linked to coumarin and fluorenone resemble donepezil and inhibit cholinesterase, offering potential for treating neurodegenerative diseases. The inherent flexibility of the indole structure, along with its ability to target multiple biological pathways, continues to inspire new drug designs, paving the way for next-generation treatments. The successful development of these compounds into clinical candidates will require a careful balance of optimizing their effectiveness, safety, and pharmacokinetics. As research in this area progresses, indole derivatives are likely to play an increasingly important role in advancing therapeutic strategies, impacting the future of drug discovery.

## Figures and Tables

**Table 1:** Examples of FDA-approved drugs containing indole structures and their therapeutic applications.

Drug Name	Chemical Structure	Indication	Mechanism of Action
**Pindolol**	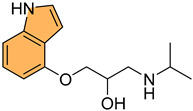	Hypertension, angina pectoris [24]	Non-selective beta-blockers decrease heart rate and blood pressure by blocking beta-1 and beta-2 adrenergic receptors [24].
**Carvedilol**	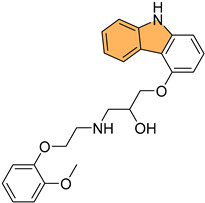	Hypertension, heart failure, left ventricular dysfunction post-myocardial infarction [25]	Non-selective beta-blockers and alpha-1 blockers reduce heart rate, blood pressure, and myocardial oxygen demand [25].
**Oxitriptan**	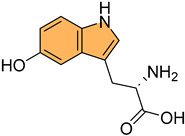	Depression, sleep disorders, appetite suppression [26,27]	Precursor to serotonin; increases serotonin levels in the brain [26].
**Sumatriptan**	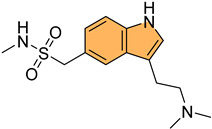	Migraine, cluster headaches [28]	Selective serotonin receptor agonist (5-HT1B/1D) causes vasoconstriction and reduces neurogenic inflammation [28].
**Panobinostat**	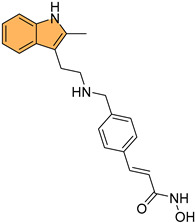	Multiple myeloma [29]	Histone deacetylase inhibitor induces cell cycle arrest and apoptosis in cancer cells [29].
**Tropisetron**	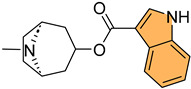	Nausea and vomiting induced by chemotherapy or surgery [30]	5-HT3 receptor antagonist blocks serotonin receptors in the central nervous and gastrointestinal tract [30].
**Pergolide**	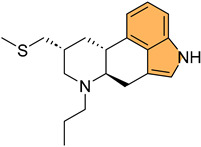	Parkinson’s disease [31]	Dopamine receptor agonists stimulate dopamine receptors in the brain [31].
**Iptacopan**	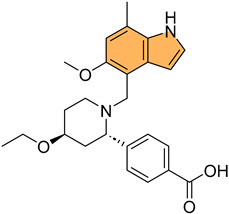	Paroxysmal nocturnal hemoglobinuria (PNH) [32]	Complement factor B inhibitor prevents the formation of the membrane attack complex [32].
**Delavirdine**	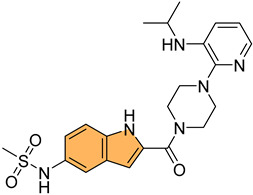	HIV-1 infection [33]	Non-nucleoside reverse transcriptase inhibitor (NNRTI) inhibits HIV-1 reverse transcriptase [33].
**Vilazodone**	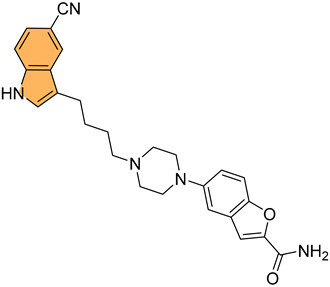	Major depressive disorder [34]	Selective serotonin reuptake inhibitor (SSRI) and partial agonist at 5-HT1A receptors [34].
**Bazedoxifene**	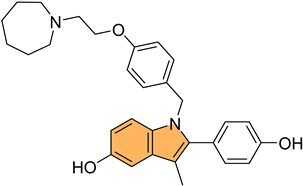	Osteoporosis in postmenopausal women [35]	Selective estrogen receptor modulator (SERM) mimics estrogen in bones to maintain bone density [35].
**Cabergoline**	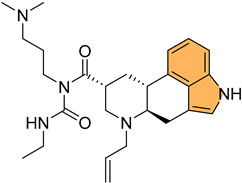	Hyperprolactinemia, Parkinson’s disease [36,37]	Dopamine receptor agonist inhibits prolactin secretion [36].
**Etodolac**	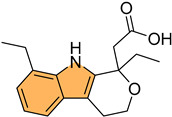	Pain and inflammation [38]	Non-steroidal anti-inflammatory drug (NSAID) inhibits cyclooxygenase (COX) enzymes, reducing prostaglandin synthesis [38].

## References

[1] Smith B.J., Liu R. (1999). A theoretical investigation of indole tautomers. J. Mol. Struct. Theochem.

[2] Kumar S., Ritika (2020). A brief review of the biological potential of indole derivatives. Future J. Pharm. Sci..

[3] Singh T.P., Singh O.M. (2018). Recent Progress in Biological Activities of Indole and Indole Alkaloids. Mini Rev. Med. Chem..

[4] Li T., Xu H. (2022). Recent Progress of Bioactivities, Mechanisms of Action, Total Synthesis, Structural Modifications and Structure-activity Relationships of Indole Derivatives: A Review. Bentham Sci..

[5] Kumari A., Singh R.K. (2019). Medicinal chemistry of indole derivatives: Current to future therapeutic prospectives. Bioorganic Chem..

[6] Shamon S.D., Perez M.I. (2016). Blood pressure-lowering efficacy of reserpine for primary hypertension. Cochrane Database Syst. Rev..

[7] Hoenders H.J.R., Bartels-Velthuis A.A., Vollbehr N.K., Bruggeman R., Knegtering H., de Jong J.T.V.M. (2018). Natural Medicines for Psychotic Disorders. J. Nerv. Ment. Dis..

[8] Steiger H. (2004). Eating disorders and the serotonin connection: State, trait and developmental effects. J. Psychiatry Neurosci..

[9] Portas C.M., Bjorvatn B., Ursin R. (2000). Serotonin and the sleep/wake cycle: Special emphasis on microdialysis studies. Prog. Neurobiol..

[10] Alivisatos S.G.A., Papaphilis A.D., Ungar F., Seth P.K. (1970). Chemical Nature of Binding of Serotonin in the Central Nervous System. Nature.

[11] Ferlazzo N., Andolina G., Cannata A., Costanzo M.G., Rizzo V., Currò M., Ientile R., Caccamo D. (2020). Is Melatonin the Cornucopia of the 21st Century?. Antioxidants.

[12] Disorders S. (2022). Role of Melatonin in the Management of Sleep and Circadian Disorders in the Context of Psychiatric Illness. Curr. Psychiatry Rep..

[13] Anticancer Potential of Indole Derivatives: An Update. https://www.degruyter.com/document/doi/10.1515/psr-2021-0028/html.

[14] Kaur K., Jaitak V. (2019). Recent Development in Indole Derivatives as Anticancer Agents for Breast Cancer. Anti-Cancer Agents Med. Chem..

[15] Sunitinib in the Treatment of Renal Cell Carcinoma: An Update on Recent Evidence—PMC. https://www.ncbi.nlm.nih.gov/pmc/articles/PMC5896861/.

[16] Casali P.G., Garrett C.R., Blackstein M.E., Shah M., Verweij J., McArthur G., Judson I., Li J., Baum C.M., Demetri G.D. (2006). Updated results from a phase III trial of sunitinib in GIST patients (pts) for whom imatinib (IM) therapy has failed due to resistance or intolerance. JCO.

[17] Qin H.-L., Liu J., Fang W.-Y., Ravindar L., Rakesh K.P. (2020). Indole-based derivatives as potential antibacterial activity against methicillin-resistance *Staphylococcus aureus* (MRSA). Eur. J. Med. Chem..

[18] Selsted M.E., Novotny M.J., Morris W.L., Tang Y.Q., Smith W., Cullor J.S. (1992). Indolicidin, a novel bactericidal tridecapeptide amide from neutrophils. J. Biol. Chem..

[19] Yaikhan T., Chuerboon M., Tippayatham N., Atimuttikul N., Nuidate T., Yingkajorn M., Tun A.W., Buncherd H., Tansila N. (2019). Indole and Derivatives Modulate Biofilm Formation and Antibiotic Tolerance of *Klebsiella pneumoniae*. Indian J. Microbiol..

[20] Nalamachu S., Wortmann R. (2014). Role of indomethacin in acute pain and inflammation management: A review of the literature. Postgrad. Med..

[21] Ahmad A., Biersack B., Li Y., Kong D., Bao B., Schobert R., Padhye S.B., Sarkar F.H. Targeted Regulation of PI3K/Akt/mTOR/NF-κB Signaling by Indole Compounds and their Derivatives: Mechanistic Details and Biological Implications for Cancer Therapy. https://www.eurekaselect.com/article/54078.

[22] Design, Synthesis, Biological Evaluation and Docking Study of Novel Indole-2-Amide as Anti-Inflammatory Agents with Dual Inhibition of COX and 5-LOX—ScienceDirect. https://www.sciencedirect.com/science/article/pii/S0223523419306257.

[23] Philoppes J.N., Abdelgawad M.A., Abourehab M.A.S., Sebak M., Darwish M.A., Lamie P.F. (2023). Novel N-methylsulfonyl-indole derivatives: Biological activity and COX-2/5-LOX inhibitory effect with improved gastro protective profile and reduced cardio vascular risks. J. Enzyme Inhib. Med. Chem..

[24] Golightly L.K. (1982). Pindolol: A review of its pharmacology, pharmacokinetics, clinical uses, and adverse effects. Pharmacotherapy.

[25] Ruffolo R.R., Feuerstein G.Z. (1997). Pharmacology of carvedilol: Rationale for use in hypertension, coronary artery disease, and congestive heart failure. Cardiovasc. Drugs Ther..

[26] Shaw K., Turner J., Del Mar C. (2002). Tryptophan and 5-Hydroxytryptophan for depression. Cochrane Database Syst. Rev..

[27] Hendricks E.J. (2017). Off-label drugs for weight management. DMSO.

[28] Ferrari M.D., Saxena P.R. (1992). Clinical effects and mechanism of action of sumatriptan in migraine. Clin. Neurol. Neurosurg..

[29] Moore D. (2016). Panobinostat (Farydak): A Novel Option for the Treatment of Relapsed or Relapsed and Refractory Multiple Myeloma. Pharm. Ther..

[30] Simpson K., Spencer C.M., McClellan K.J. (2000). Tropisetron: An update of its use in the prevention of chemotherapy-induced nausea and vomiting. Drugs.

[31] Langtry H.D., Clissold S.P. (1990). Pergolide. A review of its pharmacological properties and therapeutic potential in Parkinson’s disease. Drugs.

[32] Xu B., Kang B., Chen J., Li S., Zhou J. (2024). Factor B inhibitor iptacopan for the treatment of paroxysmal nocturnal hemoglobinuria. Blood Rev..

[33] Scott L.J., Perry C.M. (2000). Delavirdine: A review of its use in HIV infection. Drugs.

[34] Wang S.-M., Han C., Lee S.-J., Patkar A.A., Masand P.S., Pae C.-U. (2016). Vilazodone for the Treatment of Depression: An Update. Chonnam. Med. J..

[35] Yavropoulou M.P., Makras P., Anastasilakis A.D. (2019). Bazedoxifene for the treatment of osteoporosis. Expert Opin. Pharmacother..

[36] Rains C.P., Bryson H.M., Fitton A. (1995). Cabergoline. A review of its pharmacological properties and therapeutic potential in the treatment of hyperprolactinaemia and inhibition of lactation. Drugs.

[37] Curran M.P., Perry C.M. (2004). Cabergoline: A review of its use in the treatment of Parkinson’s disease. Drugs.

[38] Bellamy N. (1997). Etodolac in the management of pain: A clinical review of a multipurpose analgesic. Inflammopharmacology.

[39] Latest Global Cancer Data: Cancer Burden Rises to 19.3 Million New Cases and 10.0 Million Cancer Deaths in 2020. https://www.iarc.who.int/news-events/latest-global-cancer-data-cancer-burden-rises-to-19-3-million-new-cases-and-10-0-million-cancer-deaths-in-2020.

[40] Hashem H., Hassan A., Abdelmagid W.M., Habib A.G.K., Abdel-Aal M.A.A., Elshamsy A.M., El Zawily A., Radwan I.T., Bräse S., Abdel-Samea A.S. (2024). Synthesis of New Thiazole-Privileged Chalcones as Tubulin Polymerization Inhibitors with Potential Anticancer Activities. Pharmaceuticals.

[41] Al-Wahaibi L.H., Elshamsy A.M., Ali T.F.S., Youssif B.G.M., Bräse S., Abdel-Aziz M., El-Koussi N.A. (2024). Design and Synthesis of New Dihydropyrimidine Derivatives with a Cytotoxic Effect as Dual EGFR/VEGFR-2 Inhibitors. ACS Omega.

[42] Mohammed H.H.H., El-Hafeez A.A.A., Abbas S.H., Abdelhafez E.-S.M.N., Abuo-Rahma G.E.-D.A. (2016). New antiproliferative 7-(4-(N-substituted carbamoylmethyl)piperazin-1-yl) derivatives of ciprofloxacin induce cell cycle arrest at G2/M phase. Bioorg. Med. Chem..

[43] Mohammed H.H.H., Abbas S.H., Hayallah A.M., Abuo-Rahma G.E.-D.A., Mostafa Y.A. (2021). Novel urea linked ciprofloxacin-chalcone hybrids having antiproliferative topoisomerases I/II inhibitory activities and caspases-mediated apoptosis. Bioorg. Chem..

[44] Mohammed H.H.H., El-Hafeez A.A.A., Ebeid K., Mekkawy A.I., Abourehab M.A.S., Wafa E.I., Alhaj-Suliman S.O., Salem A.K., Ghosh P., Abuo-Rahma G.E.-D.A. (2022). New,3-triazole linked ciprofloxacin-chalcones induce DNA damage by inhibiting human topoisomerase I& II and tubulin polymerization. J. Enzym. Inhib. Med. Chem..

[45] Alhaj-Suliman S.O., Naguib Y.W., Wafa E.I., Saha S., Ebeid K., Meng X., Mohammed H.H., Abuo-Rahma G.E.-D.A., Yang S., Salem A.K. (2023). A ciprofloxacin derivative with four mechanisms of action overcomes paclitaxel resistance in p53-mutant and MDR1 gene-expressing type II human endometrial cancer. Biomaterials.

[46] Al-Hakkani M.F., Ahmed N., Abbas A.A., Hassan M.H.A., Aziz H.A., Elshamsy A.M., Khalifa H.O., Abdelshakour M.A., Saddik M.S., Elsayed M.M.A. (2023). Synthesis, Physicochemical Characterization using a Facile Validated HPLC Quantitation Analysis Method of 4-Chloro-phenylcarbamoyl-methyl Ciprofloxacin and Its Biological Investigations. Int. J. Mol. Sci..

[47] Naguib Y.W., Alhaj-Suliman S.O., Wafa E.I., Saha S., Ebeid K., Mohammed H.H.H., Abdel-Rahman S.A., Abuo-Rahma G.E.-D.A., Geary S.M., Salem A.K. (2023). Ciprofloxacin Derivative-Loaded Nanoparticles Synergize with Paclitaxel Against Type II Human Endometrial Cancer. Small.

[48] Indole-Based Tubulin Inhibitors: Binding Modes and SARs Investigations—PMC. https://www.ncbi.nlm.nih.gov/pmc/articles/PMC8911766/.

[49] Kinase Inhibitor Indole Derivatives as Anticancer Agents: A Patent Review—PubMed. https://pubmed.ncbi.nlm.nih.gov/27697069/.

[50] A Detail Survey and Analysis of Selectivity Criteria for Indole-Based Histone Deacetylase 8 (HDAC8) Inhibitors—ScienceDirect. https://www.sciencedirect.com/science/article/pii/S0022286022016192.

[51] Recent Advances of Tubulin Inhibitors Targeting the Colchicine Binding Site for Cancer Therapy—PubMed. https://pubmed.ncbi.nlm.nih.gov/36551271/.

[52] Shuai W., Wang G., Zhang Y., Bu F., Zhang S., Miller D.D., Li W., Ouyang L., Wang Y. (2021). Recent Progress on Tubulin Inhibitors with Dual Targeting Capabilities for Cancer Therapy. J. Med. Chem..

[53] Khwaja S., Kumar K., Das R., Negi A.S. (2021). Microtubule associated proteins as targets for anticancer drug development. Bioorganic Chem..

[54] Mollinedo F., Gajate C. (2003). Microtubules, microtubule-interfering agents and apoptosis. Apoptosis.

[55] Lobert S., Vulevic B., Correia J.J. (1996). Interaction of Vinca Alkaloids with Tubulin:  A Comparison of Vinblastine, Vincristine, and Vinorelbine. Biochemistry.

[56] Hawash M., Kahraman D.C., Olgac A., Ergun S.G., Hamel E., Cetin-Atalay R., Baytas S.N. (2022). Design and synthesis of novel substituted indole-acrylamide derivatives and evaluation of their anti-cancer activity as potential tubulin-targeting agents. J. Mol. Struct..

[57] Shi L., Yang S., Chang J., Zhang Y., Liu W., Zeng J., Meng J., Zhang R., Wang C., Xing D. (2022). Design, synthesis and biological evaluation of 9-aryl-5H-pyrido[4,3-b]indole derivatives as potential tubulin polymerization inhibitors. Front. Chem..

[58] Song J., Guan Y.-F., Liu W.-B., Song C.-H., Tian X.-Y., Zhu T., Fu X.-J., Qi Y.-Q., Zhang S.-Y. (2022). Discovery of novel coumarin-indole derivatives as tubulin polymerization inhibitors with potent anti-gastric cancer activities. Eur. J. Med. Chem..

[59] Yan J., Xu Y., Jin X., Zhang Q., Ouyang F., Han L., Zhan M., Li X., Liang B., Huang X. (2022). Structure modification and biological evaluation of indole-chalcone derivatives as anti-tumor agents through dual targeting tubulin and TrxR. Eur. J. Med. Chem..

[60] Ren W., Deng Y., Ward J.D., Vairin R., Bai R., Wanniarachchi H.I., Hamal K.B., Tankoano P.E., Tamminga C.S., Bueno L.M.A. (2024). Synthesis and biological evaluation of structurally diverse 6-aryl-3-aroyl-indole analogues as inhibitors of tubulin polymerization. Eur. J. Med. Chem..

[61] Reddy T.S., Rai S., Koppula S.K. (2022). Synthesis of indole-tetrazole coupled aromatic amides; *In vitro* anticancer activity, *in vitro* tubulin polymerization inhibition assay and *in silico* studies. J. Mol. Struct..

[62] Boda S., Nukala S.K., Manchal R. (2022). Synthesis of Some New Indole-1,3,4-Oxadiazole Hybrids as Tubulin Polymerization Inhibitors. Russ. J. Bioorg. Chem..

[63] Hurysz B., Evans B.A., Laryea R.N., Boyer B.E., Coburn T.E., Dexter M.S., Edwards M.A., Faulkner G.V., Huss R.L., Lafferty M.M. (2023). Synthesis, modeling, and biological evaluation of anti-tubulin indole-substituted furanones. Bioorg. Med. Chem. Lett..

[64] Liang B., Zou Q., Yu L., Wang Y., Yan J., Huang B. (2023). Novel Indole-Containing Hybrids Derived from Millepachine: Synthesis, Biological Evaluation and Antitumor Mechanism Study. Molecules.

[65] Wang H., Nie C., Luo M., Bai Q., Yao Z., Lv H., Chen B., Wang J., Xu W., Wang S. (2024). Novel GSH-responsive prodrugs derived from indole-chalcone and camptothecin trigger apoptosis and autophagy in colon cancer. Bioorg. Chem..

[66] Bhullar K.S., Lagarón N.O., McGowan E.M., Parmar I., Jha A., Hubbard B.P., Rupasinghe H.P.V. (2018). Kinase-targeted cancer therapies: Progress, challenges and future directions. Mol. Cancer.

[67] Neagu M., Constantin C., Engin A.B., Engin A. (2021). Signal Transduction in Immune Cells and Protein Kinases. Protein Kinase-Mediated Decisions between Life and Death.

[68] Kannaiyan R., Mahadevan D. (2018). A comprehensive review of protein kinase inhibitors for cancer therapy. Expert. Rev. Anticancer. Ther..

[69] Zubair T., Bandyopadhyay D. (2023). Small Molecule EGFR Inhibitors as Anti-Cancer Agents: Discovery, Mechanisms of Action, and Opportunities. Int. J. Mol. Sci..

[70] Gomaa H.A.M., Shaker M.E., Alzarea S.I., Hendawy O.M., Mohamed F.A.M., Gouda A.M., Ali A.T., Morcoss M.M., Abdelrahman M.H., Trembleau L. (2022). Optimization and SAR investigation of novel 2,3-dihydropyrazino[1,2-a]indole-1,4-dione derivatives as EGFR and BRAFV600E dual inhibitors with potent antiproliferative and antioxidant activities. Bioorg. Chem..

[71] Shawish I., Nafie M.S., Barakat A., Aldalbahi A., Al-Rasheed H.H., Ali M., Alshaer W., Al Zoubi M., Al Ayoubi S., De la Torre B.G. (2022). Pyrazolyl-s-triazine with indole motif as a novel of epidermal growth factor receptor/cyclin-dependent kinase 2 dual inhibitors. Front. Chem..

[72] Khalilullah H., Agarwal D.K., Ahsan M.J., Jadav S.S., Mohammed H.A., Khan M.A., Mohammed S.A.A., Khan R. (2022). Synthesis and Anti-Cancer Activity of New Pyrazolinyl-Indole Derivatives: Pharmacophoric Interactions and Docking Studies for Identifying New EGFR Inhibitors. Int. J. Mol. Sci..

[73] Yu G.-X., Hu Y., Zhang W.-X., Tian X.-Y., Zhang S.-Y., Zhang Y., Yuan S., Song J. (2022). Design, Synthesis and Biological Evaluation of [1,2,4]Triazolo[1,5-a]pyrimidine Indole Derivatives against Gastric Cancer Cells MGC-803 via the Suppression of ERK Signaling Pathway. Molecules.

[74] Mebratu Y., Tesfaigzi Y. (2009). How ERK1/2 Activation Controls Cell Proliferation and Cell Death Is Subcellular Localization the Answer?. Cell Cycle.

[75] Guo Y.-J., Pan W.-W., Liu S.-B., Shen Z.-F., Xu Y., Hu L.-L. (2020). ERK/MAPK signalling pathway and tumorigenesis (Review). Exp. Ther. Med..

[76] Cho D., Mier J.W., Atkins M.B., Bukowski R.M., Figlin R.A., Motzer R.J. (2009). PI3K/Akt/mTOR Pathway. Renal Cell Carcinoma: Molecular Targets and Clinical Applications.

[77] Peng Y., Wang Y., Zhou C., Mei W., Zeng C. (2022). PI3K/Akt/mTOR Pathway and Its Role in Cancer Therapeutics: Are We Making Headway?. Front. Oncol..

[78] Qin J., Sun X., Ma Y., Cheng Y., Ma Q., Jing W., Qu S., Liu L. (2022). Design, synthesis and biological evaluation of novel 1,3,4,9-tetrahydropyrano[3,4-b]indoles as potential treatment of triple negative breast cancer by suppressing PI3K/AKT/mTOR pathway. Bioorg. Med. Chem..

[79] Tyagi R., Yadav K., Khanna A., Mishra S.K., Sagar R. (2024). Efficient synthesis of indole-chalcones based glycohybrids and their anticancer activity. Bioorg. Med. Chem..

[80] Ibrahim M.S., Farag B., Al-Humaidi J.Y., Zaki M.E.A., Fathalla M., Gomha S.M. (2023). Mechanochemical Synthesis and Molecular Docking Studies of New Azines Bearing Indole as Anticancer Agents. Molecules.

[81] Perike N., Edigi P.K., Nirmala G., Thumma V., Bujji S., Naikal P.S. (2022). Synthesis, Anticancer Activity and Molecular Docking Studies of Hybrid Molecules Containing Indole-Thiazolidinedione-Triazole Moieties. ChemistrySelect.

[82] Parthiban A., Sivasankar R., Rajdev B., Asha R.N., Jeyakumar T.C., Periakaruppan R., Naidu V. (2022). Synthesis, in vitro, *in silico* and DFT studies of indole curcumin derivatives as potential anticancer agents. J. Mol. Struct..

[83] Xu J., Dong X., Huang D.C.S., Xu P., Zhao Q., Chen B. (2023). Current Advances and Future Strategies for BCL-2 Inhibitors: Potent Weapons against Cancers. Cancers.

[84] Qian S., Wei Z., Yang W., Huang J., Yang Y., Wang J. (2022). The role of BCL-2 family proteins in regulating apoptosis and cancer therapy. Front. Oncol..

[85] Zhang Z., Hou L., Bai L., Pei J., Zhao S., Luan S., Liu D., Huang M., Zhao L. (2022). Discovery and structure-activity relationship studies of novel Bcl-2/Mcl-1 dual inhibitors with indole scaffold. Bioorg. Chem..

[86] Liu Y., Li J., Zhou G., Zhang J., Teng Y., Bai Z., Liu T. (2023). Design, synthesis and anticancer activity studies of novel indole derivatives as Bcl-2/Mcl-1 dual inhibitors. Med. Chem. Res..

[87] Almehdi A.M., Soliman S.S.M., El-Shorbagi A.-N.A., Westwell A.D., Hamdy R. (2023). Design, Synthesis, and Potent Anticancer Activity of Novel Indole-Based Bcl-2 Inhibitors. Int. J. Mol. Sci..

[88] Hassan M.I., Shajee B., Waheed A., Ahmad F., Sly W.S. (2013). Structure, function and applications of carbonic anhydrase isozymes. Bioorg. Med. Chem..

[89] Hashem H.H.H. (2024). Design, synthesis, and molecular docking of novel urea linked 1,2,3-triazole-benzenesulfonamide hybrid as potential carbonic anhydrase inhibitors. J. Adv. Biomed. Pharm. Sci..

[90] Pastorekova S., Gillies R.J. (2019). The role of carbonic anhydrase IX in cancer development: Links to hypoxia, acidosis, and beyond. Cancer Metastasis Rev..

[91] Lee S.-H., Griffiths J.R. (2020). How and Why Are Cancers Acidic? Carbonic Anhydrase IX and the Homeostatic Control of Tumour Extracellular pH. Cancers.

[92] Kopecka J., Campia I., Jacobs A., Frei A.P., Ghigo D., Wollscheid B., Riganti C. (2015). Carbonic anhydrase XII is a new therapeutic target to overcome chemoresistance in cancer cells. Oncotarget.

[93] Kciuk M., Gielecińska A., Mujwar S., Mojzych M., Marciniak B., Drozda R., Kontek R. (2022). Targeting carbonic anhydrase IX and XII isoforms with small molecule inhibitors and monoclonal antibodies. J. Enzym. Inhib. Med. Chem..

[94] Krasavin M., Kalinin S., Sharonova T., Supuran C.T. (2020). Inhibitory activity against carbonic anhydrase IX and XII as a candidate selection criterion in the development of new anticancer agents. J. Enzym. Inhib. Med. Chem..

[95] Singh P., Goud N.S., Swain B., Sahoo S.K., Choli A., Angeli A., Kushwah B.S., Yaddanapudi V.M., Supuran C.T., Arifuddin M. (2022). Synthesis of a new series of quinoline/pyridine indole-3-sulfonamide hybrids as selective carbonic anhydrase IX inhibitors. Bioorganic Med. Chem. Lett..

[96] Demir-Yazıcı K., Trawally M., Bua S., Öztürk-Civelek D., Akdemir A., Supuran C.T., Güzel-Akdemir Ö. (2024). Novel 2-(hydrazinocarbonyl)-3-phenyl-1H-indole-5-sulfonamide based thiosemicarbazides as potent and selective inhibitors of tumor-associated human carbonic anhydrase IX and XII: Synthesis, cytotoxicity, and molecular modelling studies. Bioorg. Chem..

[97] Nguyen P.L., Elkamhawy A., Choi Y.H., Lee C.H., Lee K., Cho J. (2022). Suppression of Tumor Growth and Cell Migration by Indole-Based Benzenesulfonamides and Their Synergistic Effects in Combination with Doxorubicin. Int. J. Mol. Sci..

[98] Liu Y., Ma H., Yao J. (2020). ERα, A Key Target for Cancer Therapy: A Review. Onco Targets Ther..

[99] Sreenatha V., Srinivasa S.M., Prasad K.J.R. (2022). Design, synthesis, bioevaluation, DFT, docking, and molecular dynamic simulation for selected novel 1,3,4-Oxadiazole—Indole derivatives hybrid against estrogen receptor alpha. J. Mol. Struct..

[100] Kaur K., Verma H., Gangwar P., Jangid K., Dhiman M., Kumar V., Jaitak V. (2024). Design, synthesis, in silico and biological evaluation of new indole based oxadiazole derivatives targeting estrogen receptor alpha. Bioorg. Chem..

[101] Hu C.-J., Wang L.-Y., Chodosh L.A., Keith B., Simon M.C. (2003). Differential Roles of Hypoxia-Inducible Factor 1α (HIF-1α) and HIF-2α in Hypoxic Gene Regulation. Mol. Cell Biol..

[102] Qannita R.A., Alalami A.I., Harb A.A., Aleidi S.M., Taneera J., Abu-Gharbieh E., El-Huneidi W., Saleh M.A., Alzoubi K.H., Semreen M.H. (2024). Targeting Hypoxia-Inducible Factor-1 (HIF-1) in Cancer: Emerging Therapeutic Strategies and Pathway Regulation. Pharmaceuticals.

[103] Bui B.P., Nguyen P.L., Lee K., Cho J. (2022). Hypoxia-Inducible Factor-1: A Novel Therapeutic Target for the Management of Cancer, Drug Resistance, and Cancer-Related Pain. Cancers.

[104] Keskin S., Doğan Ş.D., Gündüz M.G., Aleksic I., Vojnovic S., Lazic J., Nikodinovic-Runic J. (2022). Indole-based hydrazone derivatives: Synthesis; cytotoxicity assessment, and molecular modeling studies. J. Mol. Struct..

[105] Rahman A., Prashanth N., Nippu B.N., Kumaraswamy H.M., Rajeshwara A.N., Satyanarayan N.D. (2022). Synthesis and anticancer screening of some novel Pd-catalysed 3-methyl indole based analogues on Mia PaCa-2 cell line. J. Mol. Struct..

[106] Ropero S., Esteller M. (2007). The role of histone deacetylases (HDACs) in human cancer. Mol. Oncol..

[107] Liang T., Wang F., Elhassan R.M., Cheng Y., Tang X., Chen W., Fang H., Hou X. (2023). Targeting histone deacetylases for cancer therapy: Trends and challenges. Acta Pharm. Sin. B.

[108] Jiang B.-E., Hu J., Liu H., Liu Z., Wen Y., Liu M., Zhang H.-K., Pang X., Yu L.-F. (2022). Design, synthesis, and biological evaluation of indole-based hydroxamic acid derivatives as histone deacetylase inhibitors. Eur. J. Med. Chem..

[109] Kim D., Kim K.I., Baek S.H. (2021). Roles of lysine-specific demethylase 1 (LSD1) in homeostasis and diseases. J. Biomed. Sci..

[110] Yang F.-F., Xu X.-L., Hu T., Liu J.-Q., Zhou J.-Z., Ma L.-Y., Liu H.-M. (2023). Lysine-Specific Demethylase 1 Promises to Be a Novel Target in Cancer Drug Resistance: Therapeutic Implications. J. Med. Chem..

[111] Zhang X., Sun Y., Huang H., Wang X., Wu T., Yin W., Li X., Wang L., Gu Y., Zhao D. (2022). Identification of novel indole derivatives as highly potent and efficacious LSD1 inhibitors. Eur. J. Med. Chem..

[112] Hedstrom L. (2009). IMP Dehydrogenase: Structure, Mechanism and Inhibition. Chem. Rev..

[113] Naffouje R., Grover P., Yu H., Sendilnathan A., Wolfe K., Majd N., Smith E.P., Takeuchi K., Senda T., Kofuji S. (2019). Anti-Tumor Potential of IMP Dehydrogenase Inhibitors: A Century-Long Story. Cancers.

[114] Jia H.-W., Yang H.-L., Xiong Z.-L., Deng M.-H., Wang T., Liu Y., Cheng M. (2022). Design, synthesis and antitumor activity evaluation of novel indole acrylamide derivatives as IMPDH inhibitors. Bioorg. Chem..

[115] Lee C.G., Park G.-Y., Han Y.K., Lee J.H., Chun S.H., Park H.-Y., Lim K.-H., Kim E.-G., Choi Y.-J., Yang K. (2013). Roles of 14-3-3η in mitotic progression and its potential use as a therapeutic target for cancers. Oncogene.

[116] Aseervatham J. (2022). Interactions between 14-3-3 Proteins and Actin Cytoskeleton and Its Regulation by microRNAs and Long Non-Coding RNAs in Cancer. Endocrines.

[117] Gao Z., Fan T., Chen L., Yang M., Wong V.K.W., Chen D., Liu Z., Zhou Y., Wu W., Qiu Z. (2022). Design, synthesis and antitumor evaluation of novel 1H-indole-2-carboxylic acid derivatives targeting 14-3-3η protein. Eur. J. Med. Chem..

[118] Yao C.-H., Wu M.-H., Chang P.-W., Wu S.-H., Song J.-S., Huang H.-H., Chen Y.-C., Lee J.-C. (2024). Design, synthesis, and anticancer evaluation of 1-benzo[1,3]dioxol-5-yl-3-N-fused heteroaryl indoles. Mol. Divers.

[119] Deng H., Huang M., Liu H., Zhang H., Liu L., Gao B., Li X., Li J., Niu Q., Zhang Z. (2022). Development of a series of novel Mcl-1 inhibitors bearing an indole carboxylic acid moiety. Bioorg. Chem..

[120] Qin J., Chen X., Liu W., Chen J., Liu W., Xia Y., Li Z., Li M., Wang S., Yuan Q. (2022). Discovery of 5-((4-(pyridin-3-yl)pyrimidin-2-yl)amino)-1H-indole-2-carboxamide derivatives as novel anti-cancer agents targeting Nur77. Eur. J. Med. Chem..

[121] Guidetti L., Castelli R., Zappia A., Ferrari F.R., Giorgio C., Barocelli E., Pagliaro L., Vento F., Roti G., Scalvini L. (2024). Discovery of a new 1-(phenylsulfonyl)-1H-indole derivative targeting the EphA2 receptor with antiproliferative activity on U251 glioblastoma cell line. Eur. J. Med. Chem..

[122] Ramle A.Q., Chan N.N.M.Y., Ng M.P., Tan C.H., Sim K.S., Tiekink E.R.T., Fei C.C. (2024). Structural insights and cytotoxicity evaluation of benz[e]indole pyrazolyl-substituted amides. Mol. Divers.

[123] Du B., Liu X., Luan X., Zhang W., Zhuang C. (2023). Structure optimization of an F-indole-chalcone (FC116) on 4-methoxyphenyl group and therapeutic potential against colorectal cancers with low cytotoxicity. Bioorg. Chem..

[124] Veeranna D., Ramdas L., Ravi G., Bujji S., Thumma V., Ramchander J. (2022). Synthesis of 1,2,3-Triazole Tethered Indole Derivatives: Evaluation of Anticancer Activity and Molecular Docking Studies. ChemistrySelect.

[125] Gaur A., Peerzada M.N., Khan N.S., Ali I., Azam A. (2022). Synthesis and Anticancer Evaluation of Novel Indole Based Arylsulfonylhydrazides against Human Breast Cancer Cells. ACS Omega.

[126] Seung K.J., Keshavjee S., Rich M.L. (2015). Multidrug-Resistant Tuberculosis and Extensively Drug-Resistant Tuberculosis. Cold Spring Harb. Perspect. Med..

[127] Mohammed H.H.H., Abbas S.H., Abdelhafez E.-S.M.N., Berger J.M., Mitarai S., Arai M., Abuo-Rahma G.E.-D.A.A. (2019). Synthesis, molecular docking, antimicrobial evaluation, and DNA cleavage assay of new thiadiazole/oxadiazole ciprofloxacin derivatives. Monatsh. Chem..

[128] Mohammed H.H.H., Abuo-Rahma G.E.-D.A.A., Abbas S.H., Abdelhafez E.-S.M.N. (2019). Current Trends and Future Directions of Fluoroquinolones. Curr. Med. Chem..

[129] Mohammed H.H.H., Abdelhafez E.-S.M.N., Abbas S.H., Moustafa G.A.I., Hauk G., Berger J.M., Mitarai S., Arai M., El-Baky R.M.A., Abuo-Rahma G.E.-D.A. (2019). Design, synthesis and molecular docking of new N-4-piperazinyl ciprofloxacin-triazole hybrids with potential antimicrobial activity. Bioorganic Chem..

[130] Dewangan R.P., Singh M., Ilic S., Tam B., Akabayov B. (2021). Cell-penetrating peptide conjugates of indole-3-acetic acid-based DNA primase/Gyrase inhibitors as potent anti-tubercular agents against planktonic and biofilm culture of *Mycobacterium smegmatis*. Chem. Biol. Drug Des..

[131] Reddyrajula R., Etikyala U., Manga V., Dalimba U.K. (2024). Discovery of 1,2,3-triazole incorporated indole-piperazines as potent antitubercular agents: Design, synthesis, in vitro biological evaluation, molecular docking and ADME studies. Bioorg. Med. Chem..

[132] Bhakhar K.A., Vaghela P.V., Varakala S.D., Chudasma S.J., Gajjar N.D., Nagar P.R., Sriram D., Dhameliya T.M. (2022). Indole-2-carboxamides as New Anti-Mycobacterial Agents: Design, Synthesis, Biological Evaluation and Molecular Modeling against mmpL3. ChemistrySelect.

[133] Etchart R.J., Rambo R.S., Abbadi B.L., Sperotto N., Neves C.E., Silva F.F., Dornelles M., Duarte L., Macchi F.S., Perelló M.A. (2021). Synthesis and Antimycobacterial Activity of 3-Phenyl-1H-indoles. Molecules.

[134] Leena S.S., Kaul G., Akhir A., Saxena D., Chopra S., Deepthi A. (2022). Green synthesis and antibacterial evaluation of spiro fused tryptanthrin-thiopyrano[2,3-b]indole hybrids targeting drug-resistant *S. aureus*. Bioorg. Chem..

[135] Kuzovlev A.S., Zybalov M.D., Golovin A.V., Gureev M.A., Kasatkina M.A., Biryukov M.V., Belik A.R., Silonov S.A., Yunin M.A., Zigangirova N.A. (2023). Naphthyl-Substituted Indole and Pyrrole Carboxylic Acids as Effective Antibiotic Potentiators-Inhibitors of Bacterial Cystathionine γ-Lyase. Int. J. Mol. Sci..

[136] Zhang Z., Li W., Wu H., Liu Z., Huang H. (2023). Novel photoactivated Indole-pyridine chemotherapeutics with strong antimicrobial and antibiofilm activity toward *Staphylococcus aureus*. Bioorg. Chem..

[137] Potapov K.V., Novikov R.A., Novikov M.A., Solyev P.N., Tomilov Y.V., Kochetkov S.N., Makarov A.A., Mitkevich V.A. (2023). Synthesis of the Indole-Based Inhibitors of Bacterial Cystathionine γ-Lyase NL1-NL3. Molecules.

[138] Li Y., Kowah J.A.H., Jiang M., Wu Y., Wang L., Yang F. (2024). Synthesis, antibacterial activity, and 3D-QASR studies of matrine-indole derivatives as potential antibiotics. Bioorganic Med. Chem. Lett..

[139] Kalatuwawege I.P., Gunaratna M.J., Udukala D.N. (2021). Synthesis, In Silico Studies, and Evaluation of Syn and Anti Isomers of N-Substituted Indole-3-carbaldehyde Oxime Derivatives as Urease Inhibitors against Helicobacter pylori. Molecules.

[140] Ma J., Jiang Y., Zhuang X., Chen H., Shen Y., Mao Z., Rao G., Wang R. (2022). Discovery of novel indole and indoline derivatives against *Candida albicans* as potent antifungal agents. Bioorganic Med. Chem. Lett..

[141] Wu Y., Sun A., Chen F., Zhao Y., Zhu X., Zhang T., Ni G., Wang R. (2024). Synthesis, structure-activity relationship and biological evaluation of indole derivatives as anti-Candida albicans agents. Bioorg. Chem..

[142] Kronenberger T., Laufer S.A., Pillaiyar T. (2023). COVID-19 therapeutics: Small-molecule drug development targeting SARS-CoV-2 main protease. Drug Discov. Today.

[143] Girgis A.S., Panda S.S., Kariuki B.M., Bekheit M.S., Barghash R.F., Aboshouk D.R. (2023). Indole-Based Compounds as Potential Drug Candidates for SARS-CoV-2. Molecules.

[144] Verzola M.M.S.A., de Almeida Marques D.P., da Silva E.B., Serafim M.S.M., Ferreira R.S., Fajtová P., Kohlhoff M., O’Donoghue A.J., Maltarollo V.G., Coelho-dos-Reis J.G.A. (2023). Synthesis of indole-based ferulic acid derivatives and in vitro evaluation of antiviral activity against SARS-CoV-2. Med. Chem. Res..

[145] Soleymani N., Ahmadi S., Shiri F., Almasirad A. (2023). QSAR and molecular docking studies of isatin and indole derivatives as SARS 3CLpro inhibitors. BMC Chem..

[146] Jayabal K., Elumalai D., Leelakrishnan S., Bhattacharya S., Rengarajan V., Kannan T., Chuang S.-C. (2022). Green and Regioselective Approach for the Synthesis of 3-Substituted Indole Based 1,2-Dihydropyridine and Azaxanthone Derivatives as a Potential Lead for SARS-CoV-2 and Delta Plus Mutant Virus: DFT and Docking Studies. ACS Omega.

[147] Geedkar D., Kumar A., Sharma P. (2024). Synthesis and in silico inhibitory action studies of azo-anchored imidazo[4,5-b]indole scaffolds against the COVID-19 main protease (Mpro). Sci. Rep..

[148] Zhang R.-H., Chen G.-Q., Wang W., Wang Y.-C., Zhang W.-L., Chen T., Xiong Q.-Q., Zhao Y.-L., Liao S.-G., Li Y.-J. (2024). Design, synthesis and biological evaluation of indole-2-carboxylic acid derivatives as novel HIV-1 integrase strand transfer inhibitors. RSC Adv..

[149] Wang Y.-C., Zhang W.-L., Zhang R.-H., Liu C.-H., Zhao Y.-L., Yan G.-Y., Liao S.-G., Li Y.-J., Zhou M. (2023). The Discovery of Indole-2-carboxylic Acid Derivatives as Novel HIV-1 Integrase Strand Transfer Inhibitors. Molecules.

[150] Ji K., Zhang G.-N., Zhao J.-Y., Zhu M., Wang M.-H., Wang J.-X., Cen S., Wang Y.-C., Li W.-Y. (2022). Design, synthesis, and anti-influenza A virus activity evaluation of novel indole containing derivatives of triazole. Bioorg. Med. Chem. Lett..

[151] Nisha, Singh S., Sharma N., Chandra R. (2022). The indole nucleus as a selective COX-2 inhibitor and anti-inflammatory agent (2011–2022). Org. Chem. Front..

[152] Akhtar M., Lai L., Tian T., Zhang X., Cheng H., Lin L. (2024). A series of indole-derived γ-hydroxy propiolate esters as potent anti-inflammatory agents: Design, synthesis, in-vitro and in-vivo biological studies. Eur. J. Med. Chem..

[153] Bhatia R., Vyas A., El-Bahy S.M., Hessien M.M., Mersal G.A.M., Ibrahim M.M., Dogra R., Kumar B., Design R. (2022). Synthesis, Pharmacological and In-silico Investigation of Indole-Functionalized Isoxazoles as Anti-inflammatory Agents. ChemistrySelect.

[154] Faura G.G., Wu B., Oyelere A.K., France S. (2022). Synthetic methodology-enabled discovery of a tunable indole template for COX-1 inhibition and anti-cancer activity. Bioorg. Med. Chem..

[155] Wang H., Cui E., Li J., Ma X., Jiang X., Du S., Qian S., Du L. (2022). Design and synthesis of novel indole and indazole-piperazine pyrimidine derivatives with anti-inflammatory and neuroprotective activities for ischemic stroke treatment. Eur. J. Med. Chem..

[156] Zheng Z., Li X., Chen P., Zou Y., Shi X., Li X., Kim E.Y., Liao J., Yang J., Chattipakorn N. (2023). Design and synthesis optimization of novel diimide indoles derivatives for ameliorating acute lung injury through modulation of NF-κB signaling pathway. Bioorg. Chem..

[157] Chen T., Wei Y., Zhu G., Zhao H., Zhang X. (2021). Design, synthesis and structure-activity relationship studies of 4-indole-2-arylaminopyrimidine derivatives as anti-inflammatory agents for acute lung injury. Eur. J. Med. Chem..

[158] Baramaki I., Altıntop M.D., Arslan R., Altınok F.A., Özdemir A., Dallali I., Hasan A., Türkmen N.B. (2024). Design, Synthesis, and In Vivo Evaluation of a New Series of Indole-Chalcone Hybrids as Analgesic and Anti-Inflammatory Agents. ACS Omega.

[159] Jin J., He H., Zhang X., Wu R., Gan L., Li D., Lu Y., Wu P., Wong W.-L., Zhang K. (2021). The in vitro and in vivo study of oleanolic acid indole derivatives as novel anti-inflammatory agents: Synthesis, biological evaluation, and mechanistic analysis. Bioorg. Chem..

[160] Zhu Y., Zhao J., Luo L., Gao Y., Bao H., Li P., Zhang H. (2021). Research progress of indole compounds with potential antidiabetic activity. Eur. J. Med. Chem..

[161] Ritu, Sharma P., Gupta G.D., Asati V. (2023). Design, synthesis and antidiabetic study of triazole clubbed indole derivatives as α-glucosidase inhibitors. Bioorg. Chem..

[162] Sayahi M.H., Zareei S., Halimi M., Alikhani M., Moazzam A., Mohammadi-Khanaposhtani M., Mojtabavi S., Faramarzi M.A., Rastegar H., Taslimi P. (2024). Design, synthesis, in vitro, and in silico anti-α-glucosidase assays of N-phenylacetamide-1,2,3-triazole-indole-2-carboxamide derivatives as new anti-diabetic agents. Sci. Rep..

[163] Niri D.R., Sayahi M.H., Behrouz S., Moazzam A., Mojtabavi S., Faramarzi M.A., Larijani B., Rastegar H., Mohammadi-Khanaposhtani M., Mahdavi M. (2022). Design, synthesis, in vitro, and in silico biological evaluations of coumarin-indole hybrids as new anti-α-glucosidase agents. BMC Chem..

[164] Taha M., Imran S., Salahuddin M., Iqbal N., Rahim F., Uddin N., Shehzad A., Farooq R.K., Alomari M., Khan K.M. (2021). Evaluation and docking of indole sulfonamide as a potent inhibitor of α-glucosidase enzyme in streptozotocin -induced diabetic albino wistar rats. Bioorg. Chem..

[165] Solangi M., Kanwal, Khan K., Saleem F., Hameed S., Iqbal J., Shafique Z., Qureshi U., Ul-Haq Z., Taha M. (2020). Indole acrylonitriles as potential anti-hyperglycemic agents: Synthesis, α-glucosidase inhibitory activity and molecular docking studies. Bioorganic Med. Chem..

[166] Wu P., He H., Ma H., Tu B., Li J., Guo S., Chen S., Cao N., Zheng W., Tang X. (2021). Oleanolic acid indole derivatives as novel α-glucosidase inhibitors: Synthesis, biological evaluation, and mechanistic analysis. Bioorg. Chem..

[167] Hu C., Liang B., Sun J., Li J., Xiong Z., Wang S.-H., Xuetao X. (2024). Synthesis and biological evaluation of indole derivatives containing thiazolidine-2,4-dione as α-glucosidase inhibitors with antidiabetic activity. Eur. J. Med. Chem..

[168] Taha M., Alrashedy A.S., Almandil N.B., Iqbal N., Anouar E.H., Nawaz M., Uddin N., Chigurupati S., Wadood A., Rahim F. (2021). Synthesis of indole derivatives as diabetics II inhibitors and enzymatic kinetics study of α-glucosidase and α-amylase along with their in-silico study. Int. J. Biol. Macromol..

[169] Jagadeesan S., Subramanian K., Noor A., Basu R. (2023). Indole 3-heterocyclic derivative: A potential antioxidant, antidiabetic agent and their docking study on alpha amylase. J. Mol. Struct..

[170] Tamura Y., Morita I., Hinata Y., Kojima E., Ozasa H., Ikemoto H., Asano M., Wada T., Hayasaki-Kajiwara Y., Iwasaki T. (2022). Identification of novel indole derivatives as highly potent AMPK activators with anti-diabetic profiles. Bioorg. Med. Chem. Lett..

[171] Zhao X., Yoon D.-O., Yoo J., Park H.-J. (2021). Structure-Activity Relationship Study and Biological Evaluation of 2-(Disubstituted phenyl)-indole-5-propanoic Acid Derivatives as GPR40 Full Agonists. J. Med. Chem..

[172] Coşar E., Dincel E., Demiray S., Sucularlı E., Tüccaroğlu E., Ozsoy N., Güzeldemirci N.U. (2021). Anticholinesterase activities of novel indole-based hydrazide-hydrazone derivatives: Design, synthesis, biological evaluation, molecular docking study and in silico ADME prediction. J. Mol. Struct..

[173] Alım Z., Shirinzadeh H., Kılınç N., Dilek E., Suzen S. (2024). Assessing Indole Derivative Molecules as Dual Acetylcholinesterase and Butyrylcholinesterase Inhibitors through In Vitro Inhibition and Molecular Modelling Studies. J. Mol. Struct..

[174] Nerella A., Jeripothula M. (2021). Design, synthesis and biological evaluation of novel deoxyvasicinone-indole as multi-target agents for Alzheimer’s disease. Bioorg. Med. Chem. Lett..

[175] Nadeem M.S., Khan J.A., Kazmi I., Rashid U. (2022). Design, Synthesis, and Bioevaluation of Indole Core Containing 2-Arylidine Derivatives of Thiazolopyrimidine as Multitarget Inhibitors of Cholinesterases and Monoamine Oxidase A/B for the Treatment of Alzheimer Disease. ACS Omega.

[176] Banoo R., Nuthakki V.K., Wadje B.N., Sharma A., Bharate S.B. (2024). Design, synthesis, and pharmacological evaluation of indole-piperidine amides as Blood-brain barrier permeable dual cholinesterase and β-secretase inhibitors. Eur. J. Med. Chem..

[177] Wichur T., Pasieka A., Godyń J., Panek D., Góral I., Latacz G., Honkisz-Orzechowska E., Bucki A., Siwek A., Głuch-Lutwin M. (2021). Discovery of 1-(phenylsulfonyl)-1H-indole-based multifunctional ligands targeting cholinesterases and 5-HT6 receptor with anti-aggregation properties against amyloid-beta and tau. Eur. J. Med. Chem..

[178] Neshat N., Hashmi Z., Islam R., Aaghaz S., Das S., Sharma K., Ansari M., Alam M., Shaquiquzzaman M., Ansari M. (2024). Indole-based heterocyclic conjugates: Design, synthesis, in silico studies and cholinesterase inhibition. J. Mol. Struct..

[179] Cetin A., Toptas M., Türkan F. (2022). Synthesis, biological evaluation, and bioinformatics analysis of indole analogs on AChE and GST activities. Med. Chem. Res..

[180] Khan S., Taha M., Rahim F., Shah M., Ullah H., Bahadur A., Alrbyawi H., Dera A., Alahmdi M., Pashameah R. (2022). Synthesis, In Vitro Biological Evaluation and In Silico Molecular Docking Studies of Indole Based Thiadiazole Derivatives as Dual Inhibitor of Acetylcholinesterase and Butyrylchloinesterase. Molecules.

[181] Zhou L.-C., Liang Y.-F., Huang Y., Yang G.-X., Zheng L., Sun J.-M., Li Y., Zhu F.-L., Qian H.-W., Wang R. (2021). Design, Synthesis, and Biological Evaluation of Diosgenin-indole Derivatives as Dual-functional Agents for the Treatment of Alzheimer’s Disease. Eur. J. Med. Chem..

[182] Chiu Y.-J., Lin C.-H., Lin C.-Y., Yang P.-N., Lo Y.-S., Chen Y.-C., Chen C.-M., Wu Y.-R., Yao C.-F., Chang K.-H. (2023). Investigating Therapeutic Effects of Indole Derivatives Targeting Inflammation and Oxidative Stress in Neurotoxin-Induced Cell and Mouse Models of Parkinson’s Disease. Int. J. Mol. Sci..

[183] Liang T., Xie Z., Dang B., Wang J., Zhang T., Luan X., Lu T., Cao C., Chen X. (2023). Discovery of indole-piperazine derivatives as selective histone deacetylase 6 inhibitors with neurite outgrowth-promoting activities and neuroprotective activities. Bioorg. Med. Chem. Lett..

[184] Nishikawa-Shimono R., Kuwabara M., Fujisaki S., Matsuda D., Endo M., Kamitani M., Futamura A., Nomura Y., Yamaguchi-Sasaki T., Yabuuchi T. (2024). Discovery of novel indole derivatives as potent and selective inhibitors of proMMP-9 activation. Bioorg. Med. Chem. Lett..

[185] Pasha A., Khan A., Ullah S., Halim S., Alharthy R., Anwar M., Hussain J., Naseer M., Kashtoh H., Al-Harrasi A. (2024). Indole-based thiosemicarbazones for neurodegenerative diseases as prolyl oligopeptidase inhibitors. J. Mol. Struct..

[186] Yi C., Xue Y., Chen K., Wang T., Yu J., Wang Z., Jin C. (2022). Novel difluoromethyl-containing 1-((4-methoxy-3-(piperazin-1-yl)phenyl)sulfonyl)-1H-indole scaffold as potent 5-HT6R antagonists: Design, synthesis, biological evaluation, and early in vivo cognition-enhancing studies. Bioorg. Med. Chem..

[187] Yi C., Chen K., Liang H., Wang Z., Wang T., Li K., Yu J., Sun J., Jin C. (2022). Novel difluoromethylated 1-(phenylsulfonyl)-4-(piperazin-1-yl)-1H-indole derivatives as potent 5-HT6 receptor antagonist with AMDE-improving properties: Design, synthesis, and biological evaluation. Bioorg. Med. Chem..

[188] Danilenko A., Volov A., Volov N., Platonova Y., Savilov S. (2023). Design, synthesis and biological evaluation of novel indole-3-carboxylic acid derivatives with antihypertensive activity. Bioorganic Med. Chem. Lett..

[189] Baranwal G., Goodlett B., Arenaz C., Creed H., Navaneethabalakrishnan S., Rutkowski J., Alaniz R., Mitchell B. (2023). Indole Propionic Acid Increases T Regulatory Cells and Decreases T Helper 17 Cells and Blood Pressure in Mice with Salt-Sensitive Hypertension. Int. J. Mol. Sci..

